# Transcription factors and genetic circuits orchestrating the complex, multilayered response of *Clostridium acetobutylicum* to butanol and butyrate stress

**DOI:** 10.1186/1752-0509-7-120

**Published:** 2013-11-06

**Authors:** Qinghua Wang, Keerthi Prasad Venkataramanan, Hongzhan Huang, Eleftherios T Papoutsakis, Cathy H Wu

**Affiliations:** 1 Delaware Biotechnology Institute, University of Delaware, Newark, DE 19711, USA; 2 Center for Bioinformatics and Computational Biology, University of Delaware, Newark, DE 19711, USA; 3 Department of Computer and Information Sciences, University of Delaware, Newark, DE 19711, USA; 4 Department of Chemical and Biomolecular Engineering, University of Delaware, Newark, DE 19711, USA

**Keywords:** Gene expression, Protein-protein interaction, Transcriptional regulatory network (TRN), Transcription factor (TF), TF binding site (TFBS), Transcriptional regulator (TR)

## Abstract

**Background:**

Organisms of the genus *Clostridium* are Gram-positive endospore formers of great importance to the carbon cycle, human normo- and pathophysiology, but also in biofuel and biorefinery applications. Exposure of *Clostridium* organisms to chemical and in particular toxic metabolite stress is ubiquitous in both natural (such as in the human microbiome) and engineered environments, engaging both the general stress response as well as specialized programs. Yet, despite its fundamental and applied significance, it remains largely unexplored at the systems level.

**Results:**

We generated a total of 96 individual sets of microarray data examining the transcriptional changes in *C. acetobutylicum,* a model *Clostridium* organism*,* in response to three levels of chemical stress from the native metabolites, butanol and butyrate. We identified 164 significantly differentially expressed transcriptional regulators and detailed the cellular programs associated with general and stressor-specific responses, many previously unexplored. Pattern-based, comparative genomic analyses enabled us, for the first time, to construct a detailed picture of the genetic circuitry underlying the stress response. Notably, a list of the regulons and DNA binding motifs of the stress-related transcription factors were identified: two heat-shock response regulators, HrcA and CtsR; the SOS response regulator LexA; the redox sensor Rex; and the peroxide sensor PerR. Moreover, several transcriptional regulators controlling stress-responsive amino acid and purine metabolism and their regulons were also identified, including ArgR (arginine biosynthesis and catabolism regulator), HisR (histidine biosynthesis regulator), CymR (cysteine metabolism repressor) and PurR (purine metabolism repressor).

**Conclusions:**

Using an exceptionally large set of temporal transcriptional data and regulon analyses, we successfully built a STRING-based stress response network model integrating important players for the general and specialized metabolite stress response in *C. acetobutylicum*. Since the majority of the transcription factors and their target genes are highly conserved in other organisms of the *Clostridium* genus, this network would be largely applicable to other *Clostridium* organisms. The network informs the molecular basis of *Clostridium* responses to toxic metabolites in natural ecosystems and the microbiome, and will facilitate the construction of genome-scale models with added regulatory-network dimensions to guide the development of tolerant strains.

## Background

*Clostridium* organisms are endospore-forming anaerobic firmicutes important in pathogenesis, human physiology (with their notable role in the human gut microbiome [1]), the carbon cycle and biotechnological applications [2,3]. *C. acetobutylicum* is the first sequenced *Clostridium* and has evolved into a model organism for the genus. It can utilize a wide variety of substrates to produce metabolites useful as industrial chemicals and biofuels [4]. Most of these metabolites, and notably butyrate and butanol, are toxic to the cells greatly impacting their metabolism and survival [5]. Several studies have been published in the last few years aiming to understand the transcriptional and translational basis of metabolite stress response, yet the regulatory network beneath these responses remains incompletely understood at the systems level [6-14]. What transpires from the data of these studies is that the metabolite stress response includes virtually all annotated genes of the core stress program (the so-called heat-shock protein (HSP) response) but also many other large programs, including amino-acid and nucleic-acid biosynthetic pathways. Such programs are apparently underlying the adaptive response of the cells to these toxic metabolites. This adaptive response includes changes in membrane composition, such as increasing the fatty acid tail length and percentage of saturated fatty acids, which is known as homeoviscous adaptation in response to the fluidizing effects of organic solvents and acids [15-17].

Many bacterial genome sequences have been completed and successfully annotated, but most lack information on the regulatory front of gene expression [18]. Understanding the complex regulatory circuitry comprised of transcription factors (TFs) and their corresponding DNA targets, including the motifs or transcription factor binding sites (TFBSs), is a fundamental requirement for understanding the complexity of responses in natural habitats and microbiomes, but also for building systems-level molecular models. Integrated use of detailed experimental data and *in silico* analyses is necessary for reconstructing transcriptional regulatory networks (TRNs) [19], which improve our understanding of complex phenotypes and facilitate the development of novel strains using synthetic biology.

**Figure 1:**
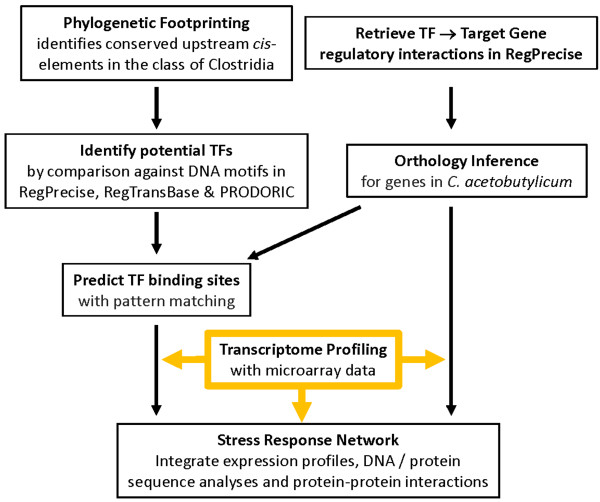
**Workflow for prediction of transcriptional regulatory interactions to reconstruct the metabolite stress response network in *C. acetobutylicum*.**

In this study, we applied a comparative-genomics, pattern-based approach to analyze a deep set of temporal transcriptional data to infer the transcriptional regulatory interactions underlying the metabolite stress response in *C. acetobutylicum* (Figure 1). Using Regulatory Sequence Analysis Tools (RSAT) [20], footprint-discovery [21] analysis (i.e., phylogenetic footprinting) was carried out, where the comparative analysis of the genomic context of *C. acetobutylicum* genes was successfully accomplished. Overrepresented oligonucleotides (words) or spaced pairs thereof (dyads) were detected and assembled into Position Weight Matrices (PWMs) [20,21]. These matrices were compared against the PWMs of known TF binding DNA motifs from three public resources: RegPrecise [22], RegTransbase [23] and PRODORIC databases [24] with the tool Tomtom [25]. The results, together with OMA (**O**rthologous **MA**trix) orthology inference in *C. acetobutylicum* for the genes available from RegPrecise [22,26], enabled the prediction of many TF binding sites (TFBSs) and functional assignment of select TFs and their target genes (TGs) in *C. acetobutylicum* (Figure 1). Together, results from these analyses enabled the construction of the stress response network involving the regulatory connections of key TFs and TGs engaged during stress.

First, we identify the common and distinct cellular responses to butanol and butyrate stress based on our comprehensive set of temporal transcriptional data. For the common stress responses, facilitated by target-gene predictions for the core HSP TFs, HrcA and CtsR, we expand the list of Class I, III & IV stress genes. For the specialized stress responses, we identify the genes and biological processes that display distinct expression patterns for each metabolite. This, coupled by a genome-scale analysis of transcriptional regulators (TRs) differentially expressed under the two stressors, led us to focus on core TFs that apparently orchestrate these stress responses. We analyzed the TFBSs and regulons of these TFs and examined the interconnectivity of their regulons thus arriving at a comprehensive picture of the transcriptional network that underlies the responses to two metabolite stresses.

## Results and discussion

### Overview: systems analysis identifies common and distinct cellular responses to butanol and butyrate stress

We use microarray analysis to examine the response of *C. acetobutylicum* to butanol and butyrate stress at mid-exponential phase (A_600_ ~ 1.0) of pH-controlled batch cultures up to 75 min post stress with 15 min intervals (0, 15, 30, 45, 60 and 75 min). For each metabolite, cultures were stressed with three different concentrations: 30 mM (low), 60 mM (medium) and 90 mM (high) of butanol, or with 30 mM (low), 40 mM (medium) and 50 mM (high) of butyrate. These stressor concentrations were selected based on previous studies [6,7,10] to obtain weak, moderate and strong stress responses. As described in the Methods section, we used a reference design to collect the data. Normalized microarray data were analyzed using significance analysis of microarrays [27] (SAM) to identify differentially expressed genes. We collected data from two biological replicates, with dye swaps for each of the 6 time points for each of the six stress conditions (butanol, butyrate; low, medium and high stress). Thus, we generated a total of 96 individual sets of stress data for each metabolite. These data constitute an extensive microarray data ensemble capturing the response of *C. acetobutylicum* to these two metabolites. The design of the microarrays based on the Agilent technology and the associated methods have been extensively validated [5,28]. This microarray technology offers extraordinary accuracy with many probes for each gene and multiple copies of each probe. Nevertheless, the expression data for a select subset of the genes (CAP0102, CAC1405, CAC3190, CAC0766 and CAC1391; see Methods section for details) were further validated using Q-RT-PCR from a third biological replicate.

Consistent with an earlier, lower resolution microarray study [6,7,10], our data show that butanol and butyrate stress responses of *C. acetobutylicum* include not only upregulation of heat shock protein (HSP) genes, but also the differential expression of more than 1,000 genes related to many distinct physiological functions and cellular programs (Additional file 1). Under butanol stress, 1,118 genes were differentially expressed (535 up, 575 down, 8 bimodal); whereas there are 1,390 differentially expressed genes for butyrate stress (710 up, 675 down, 5 bimodal). The union of the two includes 1,984 genes, for which K-means clustering results are shown for the butanol and butyrate stress (Additional file 2: Figure S1A and B), respectively.

**Figure 2:**
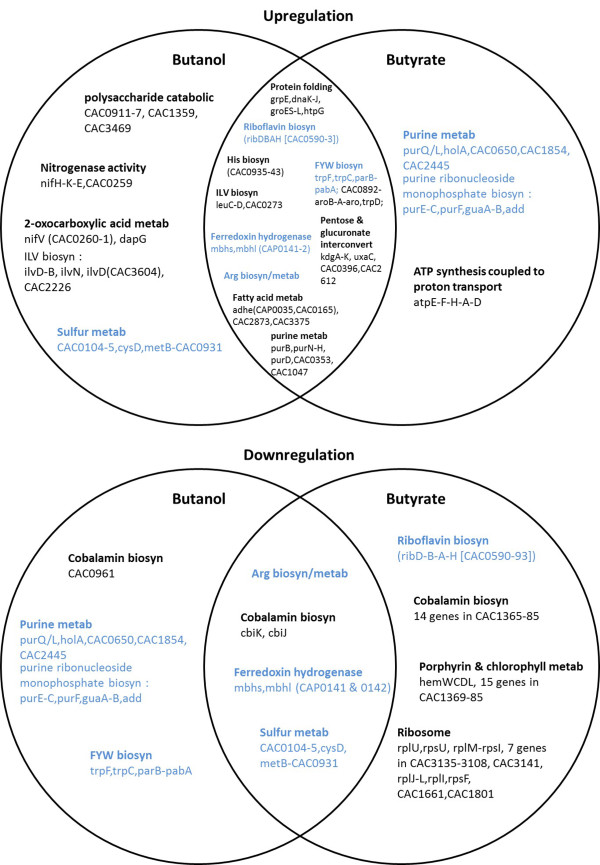
**Venn diagrams of selected genes and pathways, which were differentially upregulated or downregulated (with a fold change of >1.5 for at least 5 time points per stress experiment) in response to the butanol and butyrate stresses.** This list is not all inclusive; a complete list of differentially expressed genes is available in the Additional file 1. The genes and pathways that are both upregulated and downregulated (e.g., some genes are under bimodal regulation at a given stress, and some genes are up-regulated in one stress but down-regulated in another stress) are indicated with blue font.

Comparative analysis of the butanol (BuOH) versus butyrate (BA) stress responses is essential for understanding the general (that is, the common) stress response as well as the specialized, stressor-dependent responses. Using FIVA (Functional Information Viewer and Analyzer) [29], we identified the statistically significant differentially expressed functional categories based on annotated pathways (KEGG database [30]) and Gene Ontology (GO) annotations (from UniProtKB [31]) for the *C. acetobutylicum* genome. Each gene was assigned into one of four differentially expressed groups (up-regulation, down-regulation, bimodal and non-significant) for each stressor individually or in combination (e.g., BuOH-up/BA-up, BuOH-down/BA-down, BuOH-up/BA-down etc.). Then, the GO categories and the KEGG pathway classifications for each gene were utilized in FIVA to identify the significant functional categories enriched for each differentially expressed group. In Figure 2, the Venn diagrams display the most significant similarities and differences between the butanol and butyrate stresses.

### The common & general stress response: expanding the list of Class I, III & IV genes

**Figure 3:**
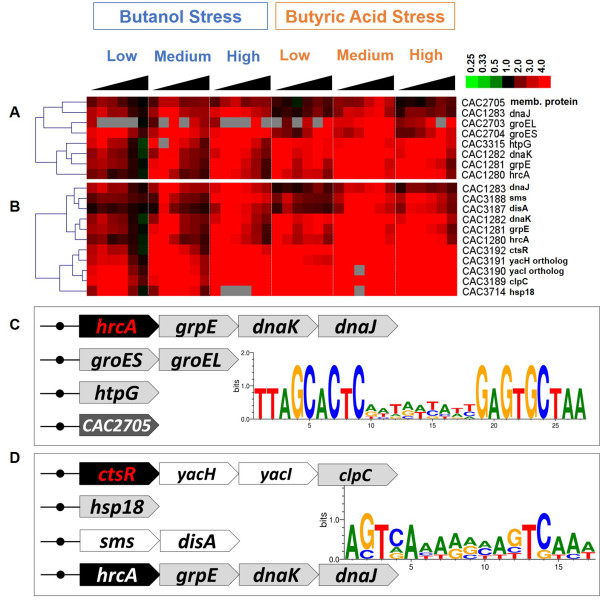
**The expression of predicted target genes for HrcA (A) and CtsR (B).** The putative regulon structure is shown for HrcA **(C)** and CtsR **(D)** (see text for details). Genes in each listed operon are shown by arrows; TF genes are in black, chaperone proteins are in light grey; potential novel genes for a regulon in *C. acetobutylicum* are in dark grey; presence of TF binding sites are indicated by black circles. The sequence logos (which represent sequence conservation) for HrcA and CtsR binding motifs in *C. acetobutylicum* are shown in **(C)** and **(D)**, were created with 6 and 5 binding sites respectively, using WebLogo [37,38]. Gene expression data are displayed as in fold change compared to no-stress control cultures at the same timepoint.

Consistent with previous findings [7,32,33], heat shock protein (HSP) genes (e.g., *grpE, dnaK-J*, *groES-EL*, *htpG*) and other general stress genes were upregulated under both stresses (Figure 2). The general stress genes are classified into four different categories based on the *Bacillus subtilis* model as Class I, II, III and IV [7,10,34]. Class I stress genes are under the control of HrcA (Figure 3A), and include *groES*, *groEL*, *grpE*, *dnaK, dnaJ* and *htpG*. Class III stress genes (Figure 3B) are under the regulation of CtsR (Class Three Stress Protein Regulator) and include *clpP*, *clpE*, *clpX* and the *clpC* operon. The operon information used in this study is based on the predicted transcriptional units by Paredes *et al.*[35]. The HrcA and CtsR regulons are described in detail in the following sections. Class II stress genes in *B. subtilis* are defined as those under the regulation of the stress-specific sigma factor, σ^B^. As there is no known ortholog for σ^B^ in *C. acetobutylicum* (or any other sequenced *Clostridium* organism [36]), Class II stress genes are considered absent in *C. acetobutylicum*. Nevertheless, based on the conservation of the σ^B^ regulated Class II stress proteins and using OMA analysis, we identified 33 proteins in *C. acetobutylicum* that are homologous to the *B. subtilis* Class II HSPs (Additional file 2: Figure S2). Among them, the only genes that were differentially upregulated (i.e., CAC3187-CAC3192) belong to Class III in *C. acetobutylicum*, and the rest show weak differential expression, thus further supporting the absence of Class II HSP regulation in *C. acetobutylicum*. Class IV stress genes are defined as the stress related genes that are not under the control of HrcA*,* σ^B^ or CtsR. 72 genes that were overexpressed under both butanol and butyrate stress (SAM analysis; fold change ≥ 1.5 at 0.05 FDR), but that they were not identified as Class I or III genes, were thus identified as Class IV stress genes in *C. acetobutylicum* (Additional file 2: Figure S3). This is a much larger list than previously identified in this organism [7,10] or any *Clostridium*. This large Class IV set includes genes from carbohydrate metabolism, histidine metabolism, the *sol*-locus genes (CAP0162-CAP0165), genes related to biosynthesis of membrane and cell-wall components, and stress responsive transcriptional regulators/factors (TRs, TFs).

### The two HSP regulons: the core of the stress response

We used a pattern-based approach combined with expression and functional profiling to infer potential TFBSs in the *C. acetobutylicum* genome, thereby predicting transcriptional regulations and reconstructing stress response network (Figure 1; see Methods for details). Successful identification of TFBSs for many *C. acetobutylicum* genes suggests good similarity of binding motifs between *Clostridium* organisms and other Gram^+^ bacteria. Among them, HrcA and CtsR are the two TFs orchestrating the core HSP response.

#### The HrcA regulon

Parts of the HrcA and CtsR regulons in *C. acetobutylicum* have been examined previously. Notably, it was shown that the *dnaKJ* and *groESL* operons are HrcA dependent [10,39], whereas *hsp18* is not [10]. Our sequence analysis indicates that phylogenetically conserved HrcA binding sites are present upstream of four operons: *hrcA-grpE-dnaK-dnaJ* (CAC1280-CAC1283), *groESL* (CAC2704-CAC2703), CAC2705 and *htpG* (CAC3315) (Figure 3A & C), with the first two corroborated by Bahl *et al.*[39]. All these operons, except for CAC2705, were highly overexpressed during the time course of all the tested butanol and butyrate stresses in this study, which is consistent with previous reports. Although its HrcA binding site is the same as for its adjacent *groESL* operon, CAC2705 shows significantly weaker overexpression than the other genes [7], thus suggesting that it is regulated by an additional TF. The function of the membrane protein CAC2705 or any of its orthologs is unknown. Overall, the function of HrcA in *C. acetobutylicum* is likely similar to that in many other well studied organisms, which is to maintain low basal levels of expression of the *dnaK*, *dnaJ*, *groESL* and *htpG* operons in the absence of stress [40]. Upon exposure to stress, these operons are no longer repressed and instead get strongly upregulated. The underlying regulatory mechanism is apparently complex because when *hrcA* transcription is upregulated, it should lead to the synthesis of more HrcA repressor protein and therefore the repression of these operons should get stronger. But this is in contrast to the fact that these operons get upregulated at the same time as *hrcA* is upregulated. In *B. subtilis*, complex post-transcriptional regulations of the *hrcA* operon, based on mRNA processing and stability, ensures the production of the operon’s proteins in the amounts needed by the cell [41]. Additionally, HrcA is activated by the free GroESL protein complex under normal conditions, but during stress, titration of GroESL by unfolded proteins constrains HrcA activation, thus ensuring additional post-translational control [42].

#### The CtsR regulon

Phylogenetic footprinting analysis of the *C. acetobutylicum* genome revealed the presence of CtsR operator sites upstream of *ctsR-yacH-yacI-clpC* (CAC3192-CAC3189) and *hsp18* (CAC3714) [7,10]. By obtaining the *C. acetobutylicum* orthologs for all the target genes of CtsR regulons in RegPrecise, we found additional putative target genes in *C. acetobutylicum*, including the operon of *sms-disA* (CAC3188-CAC3187) and *hrcA-grpE-dnaK-dnaJ* (CAC1280-CAC1283) (Figure 3B & D), whose TFBSs are less similar to CtsR DNA motifs (via Matrix Scan, see Methods section) than those of *ctsR* and *hsp18* operons. Their gene expression patterns align well with *ctsR* and *hsp18* (Figure 3B). The presence of CtsR binding site upstream of *hrcA* operon confirms the presence of secondary control of the *dnaK* operon by Homuth *et. al.* (1999) [41], leading to a cross regulation of CtsR and HrcA, which has been observed in other gram positive organisms [43]. *sms* (CAC3188) codes for an ortholog for the DNA repair protein RadA in *B. subtilis* str. 168, which may play a role in the repair of endogenous alkylation damage [44]. DisA (CAC3187) likely participates in a DNA-damage check-point that is active prior to asymmetric division when DNA is damaged, like in *B. subtilis*[45]. DisA in *B. subtilis* forms globular foci that rapidly scan along the chromosomes during sporulation, searching for lesions. When a lesion is present, DisA pauses at the lesion site. This triggers a cellular response that culminates in a temporary block in sporulation initiation [45]. CtsR, although a repressor of HSPs, was observed to have higher expression of its transcripts during the onset of stress, similar to HrcA. Repression of CtsR under stress is overcome by its inactivation through phosphorylation of arginine residues by YacI. YacI under normal conditions is bound to ClpC, but is activated under stress by YacH [46].

### Stressor-dependent stress responses

#### Overview

Many genes in the COG (Clusters of Orthologous Groups) category of amino acid transport and metabolism displayed different patterns of expression under the two stresses. Some of these genes are involved in the biosynthesis of arginine, histidine and tryptophan, and these are discussed in detail below. While some purine metabolism-related genes (*purB, purN-H, purD,* CAC0353 and CAC1047, Figure 2) were upregulated under both stresses, some of the other genes (*purE-C, purF, guaA-B, add, purQ/L, holA,* CAC0650*,* CAC1854 and CAC2445) were upregulated under butyrate stress, but downregulated under butanol stress.

Other sets of genes that display different expression patterns under the two stresses are genes involved in ATP synthesis genes, cobalamin biosynthesis, and ribosomal protein genes. As shown in Figure 2, 5 genes (i.e., *atpA, atpD, atpE, atpF* and *atpH*) (within the CAC2872-CAC2864 operon), involved in ATP synthesis coupled to proton transport, were upregulated under butyrate stress only. Nine genes involved in polysaccharide catabolic processes (CAC0911-CAC0917, CAC3469 and CAC1359) were upregulated under butanol stress only, with a few genes among them also upregulated by transient butanol pulse in chemostat cells [32]. The majority of the genes involved in cobalamin biosynthesis were down-regulated under butyrate stress but not under butanol stress, which is consistent with a previous study [47]. They include 14 genes, *cbiM, cbiG, cobT, cbiP, cobB, cbiC, cbiD, cbiT, cobI/cbiL, cbiF/cobM, cbiH/cobJ, cobU, cobS* and *cobC*, which are part of the CAC1365-CAC1386 operon. This suggests a lowered need for cobalamin for the strain under butyrate stress. Cobalamin is a necessary cofactor for various reactions involving rearrangements including glycerol dehydratases for glycerol metabolism and transmethylation for the formation of methionine from homocysteine [47]. 27 out of 48 ribosomal protein-encoding genes were downregulated under butyrate stress. Among them, 18 were only downregulated under butyrate stress but not so under butanol stress (Figure 2 and Additional file 2: Figure S4). These data suggest that translation is suppressed during butyrate stress at all three stressor levels (low, medium and high), consistent with the previous studies [7].

### Amino- and nucleic-acid biosynthesis

**Figure 4:**
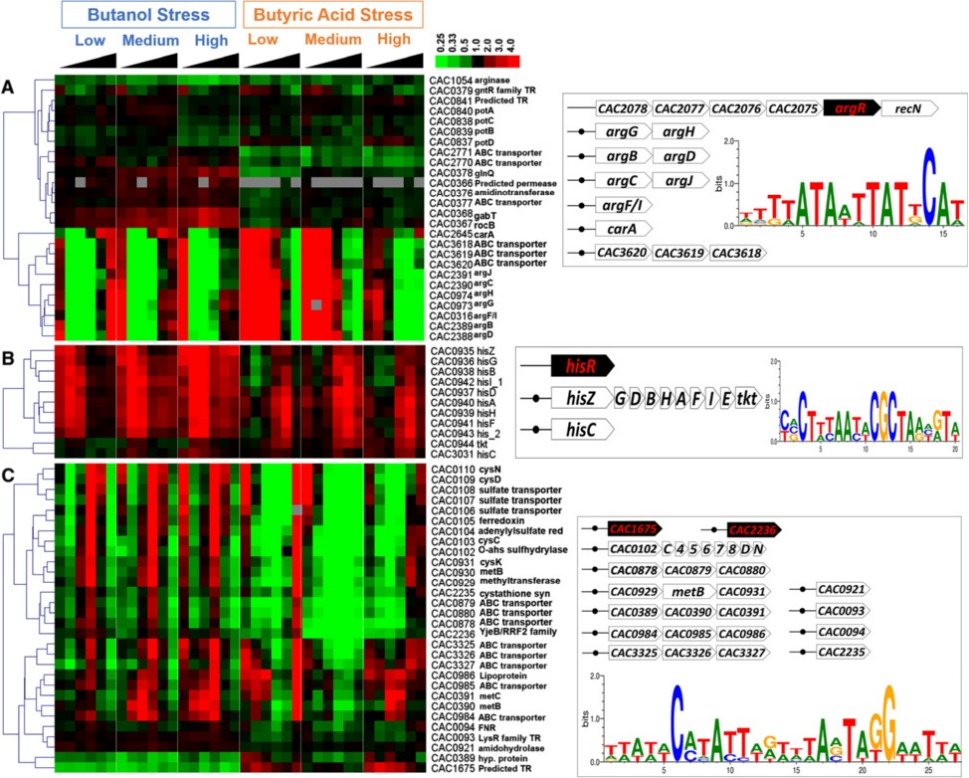
**Expression patterns of genes in the arginine and histidine biosynthesis and cysteine metabolism, and the associated transcriptional regulators. (A)** ArgR-regulated genes. **(B)** HisR-regulated genes. **(C)** CymR-regulated genes. The sequence logos shown on the right are made with 9, 3 and 8 TF binding sites, respectively.

***Arginine biosynthesis*** related genes (e.g., *argF/I* (CAC0316), *argG-argH* (CAC0973-CAC0974), *argB-argD* (CAC2389-CAC2388), *argC-argJ* (CAC2390-CAC2391), *carB* (CAC2644), *carA* (CAC2645) and CAC3619 (coding for amino acid ABC transporter component)) exhibited dose- and time-dependent expression for both butanol and butyrate stresses (Figure 4A and Additional file 2: Figure S6). For example, their expression was more strongly upregulated at the low compared to high butyrate stress. In addition, they show different expression patterns from those reported by Alsaker *et al.* in 2010 [7] when comparing the low and medium levels of butyrate stress at 30, 45, and 60 min post stress. These differences can be ascribed to the different experimental conditions used in the two studies. In the 2010 study, Clostridial Growth Medium (CGM) containing yeast extract was used without pH control in static-flask cultures, whereas in the current study, a defined medium was employed in agitated bioreactors with pH controlled at 5.0. Based on the fact that butyrate inhibition is pH dependent [7], and that pH-dependent growth play a crucial role in the cellular adaptation [48], our assessment is that these difference are predominantly due to the impact of pH, but also perhaps the presence of arginine or intermediates of its biosynthesis in the complex CGM medium. In *Escherichia coli*, and several other organisms, the arginine decarboxylase/antiporter-dependent acid-resistance (AR) system 3 (AR3) [49], is one of the four known AR systems protecting cells from acid stress. But as already discussed [50], there are no ortholog genes to these or the other AR systems in *C. acetobutylicum*. Nevertheless arginine biosynthesis and transport in combination with culture pH apparently play an important role in carboxylic acid stress.

Nine genes involved in ***histidine biosynthesis*** (*hisZ-hisG-hisD-hisB-hisH-hisA-hisF-hisI*-*hisE* (CAC0935-CAC0943) displayed complex expression patterns post stress (Figure 4B). Interestingly, induction of genes for histidine biosynthesis has recently been shown to contribute to acid tolerance in *Lactobacillus casei*[51]. In addition, a histidine decarboxylation pathway introduced in *Lactococcus lactis* improved survival to acid stress [52].

Similarly, the ***tryptophan biosynthesis*** genes in the CAC3163-CAC3157 operon (*parB*, *pabA, trpD, trpC, trpF, trpB* and *trpA*) show strong dose dependence on butanol concentration (Additional file 2: Figure S6), where low butanol stress leads to strong overexpression at the early timepoints, but mostly to downregulation for medium and high butanol stress. Under ethanol stress, tryptophan genes were reported to be upregulated and functionally tested in *Saccharomyces cerevisiae*[53]. For butyrate stress, there was upregulation for all 3 levels of stress, which is unlike the Alsaker *et al.* 2010 study, where a weak downregulation was found under butyrate stress [7]. As discussed, these differences may be related to the presence of yeast extract and the lack of pH control in the 2010 study. ***Cysteine metabolism*** genes also show a butyrate dose-dependent expression (Figure 4C & Additional file 2: Figure S6)*.*

The branched-chain amino acids (BCAAs, i.e., valine, leucine and isoleucine) are involved in the synthesis of branched chain fatty acids through the formation of α-keto acids. It has been suggested [7] that BCAAs are incorporated into membrane components to generate more rigid membrane aiming to counteract the solvation effect of toxicity solvents. Homeoviscous membrane changes involving modifications of membrane fatty acid composition to counteract the fluidizing effects of butanol and other solvents have been extensively discussed in the literature [15,16]. In *Streptococcus murants*, the BCAA aminotransferase encoded by *ilvE* was shown to protect against acid stress [54]. Thus, BCAAs may serve in multiple roles in dealing with both solvent and acid toxicity. The genes *ilvC* (CAC0091) and *leuC-leuD-leuB-ilvD-ilvB* (CAC3173-CAC3169) are involved in ***valine, leucine and isoleucine biosynthesis***. Under butanol stress (Additional file 2: Figure S6), expression of these BCAA genes is dose dependent displaying stronger upregulation at high butanol levels compared to low or medium levels. These genes display a more complex pattern under butyrate stress, with significant downregulation under high stress.

Several genes involved in ***purine metabolism*** (Additional file 2: Figure S5 and S6), including *purE- purC* (CAC1390-CAC1391), *purF-purM-purN-purH* (CAC1392-CAC1395), *purD* (CAC1396) and *purQ/purL* (CAC1655), displayed distinct expression patterns for different butanol stress levels: at low and medium levels of butanol, these genes were weakly upregulated while at high level of butanol, they were downregulated. The response of these genes to butyrate stresses was strong in this study, and displayed different kinetics and strengths compared to results from Alsaker *et al.*[7]. Here, these genes show the strongest upregulation at high butyrate stress level, which may suggest their involvement in acid resistance. Insertional mutagenesis of the *Lactococcus lactis* purine metabolism gene *guaA* led to increased acid resistance [55]. Purine metabolism also shares a large number of genes and pathway intermediates with histidine metabolism, which, as already discussed, were also found to be differentially expressed under stress. It is noteworthy that metabolic pools (and, notably, purine nucleotides) impact acid tolerance [55].

### Differential expression of transcriptional regulators: overview

Given that the two stressors elicit several common but also many distinct responses per our discussion above, we aimed next to identify the TFs that might orchestrate these responses. The expression profile for the 164 significantly differentially expressed TRs in *C. acetobutylicum* is shown in Additional file 2: Figure S7. K-means clustering of these 164 TRs identified common and distinct expression patterns for TRs under the two metabolite stresses. Clusters containing TRs that are upregulated (Additional file 2: Figure S7 A, F & I) and downregulated (Additional file 2: Figure S7 C, D & E) at the same time under both stresses can be linked to general stress response, while clusters consisting of TRs with distinct expression patterns for each stress (Additional file 2: Figure S7 B, G, H & J) are involved in orchestrating the stressor-specific response.

Because a large fraction of the aforementioned 164 TRs remain non-annotated (Additional file 3), we used phylogenetic footprinting and comparison with RegPrecise DNA motifs (Figure 1), and thus many TFBSs were successfully identified in *C. acetobutylicum,* and are shown in various figures. Many such identified TFBSs correspond to a subset of the core TFs in Bacillales. These include regulators that control the metabolism of amino acids and nitrogen (ArgR, CodY and CymR), carbohydrates (CcpA and CggR), biotin cofactor (BirA), fatty acids (FapR) and nucleotides (NrdR and PurR). These regulators also include TFs for metal homeostasis (Fur, MntR and Zur), respiration (Rex), sporulation (Spo0A), stress responses (CsoR, CtsR, HrcA, LexA and PerR), as well as the chromosomal replication initiation regulator DnaA [56]. Next, we focus on selected regulators with target genes significantly differentially expressed, per our discussion above, in butanol and/or butyrate stressed *C. acetobutylicum*. We start with the three stress-related regulators, which, unlike CtsR and HrcA, have not yet been examined at the systems level in *Clostridium*. We then discuss transcriptional regulators of amino acid and purine metabolism that appear to be part of the specialized metabolite stress response.

### The core stress-associated transcription factors engaged in the butanol and butyrate stress response

#### LexA

**Figure 5:**
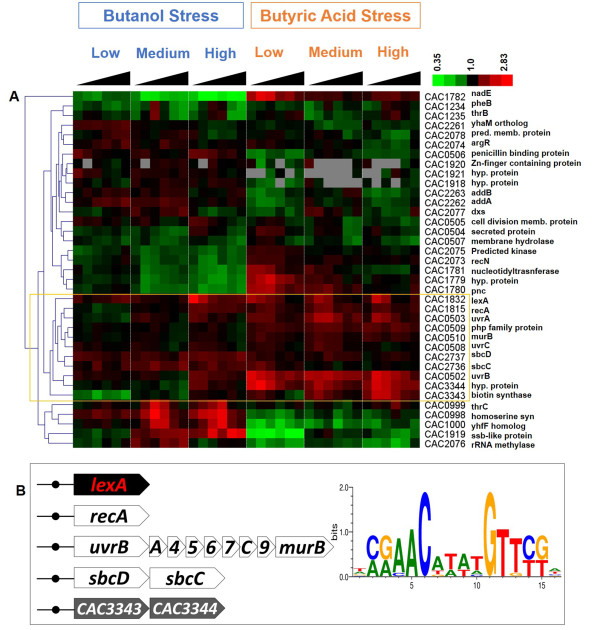
**The LexA regulon.** Expression patterns **(A)** of predicted LexA target genes, among which, those displaying differential expression similar to *lexA* and *uvrB* (inside orange box) are shown in **(B)** in operon organization together with the predicted LexA DNA binding motif (the sequence logo is made with 10 TFBSs).

LexA is well-known as the primary TF controlling the SOS response, which is an inducible DNA repair system that allows bacteria to survive sudden increases in DNA damage [57]. Even though it has been well studied in many bacteria, the LexA regulon has not been examined in *C. acetobutylicum* so far. Therefore it was important to examine which genes might be under LexA control in *C. acetobutylicum* and whether these SOS response genes are differentially regulated under butanol or butyrate stress. Butyrate stress, in particular, may cause acid-induced DNA damage, which is frequently compared to oxidative stress [58]. RSAT footprint analysis led to the identification of four genes/operons with highly conserved *cis*-elements in their promoter regions that include LexA binding sites known as SOS boxes [59]. These are *lexA* (CAC1832), *recA* (CAC1815), *uvrB-uvrA-*CAC0504-CAC0505-CAC0506-CAC0507*-uvrC-*CAC0509*-murB* (CAC0502-CAC0510), and CAC3343-CAC3344. In addition, the orthologs in *C. acetobutylicum* for all target genes in LexA regulons from RegPrecise were collected using OMA search, and scanned for LexA binding site in their operon leader sequences. The operons with an identifiable LexA binding site (p-value < 1e-3) were collected and the microarray data for their gene members are shown in Figure 5. Hierarchical clustering shows that *lexA*, *recA* and the *uvrB* operon are clustered well. This is consistent with the possibility that they are functionally correlated, like in other well studied bacteria [60,61]. Moreover, even though no ortholog of CAC3343 was listed as under LexA control in RegPrecise, our footprinting analysis together with expression data suggests that the CAC3343-CAC3344 operon is also putatively controlled by LexA. CAC3343 encodes a putative DNA modification/repair radical SAM protein and CAC3344 encodes a protein of unknown function. In addition, the operon of *sbcD-sbcC* (CAC2737-CAC2736; *sbcD*: DNA repair exonuclease; *sbcC*: ATPase involved in DNA repair) is clustered closely to *recA*, and its promoter region shows a potential LexA binding site (p-value < 1e-3). The UvrABC repair system catalyzes the recognition and processing of DNA lesions. A damage recognition complex composed of 2 UvrA and 2 UvrB subunits scans DNA for abnormalities. Regulation of the UvrABC system by LexA is well established in many bacteria, including *E. coli* and *B. subtilis*[60,61], but has not been examined in *Clostridium*. The more significant upregulation of *uvrB* under butyrate stress compared to butanol stress suggests that DNA damage may be more prevalent under butyrate stress. As discussed, butyrate stress affects the cells similar to oxidative stress by damaging DNA, proteins and lipids [58]. Therefore, we conclude that the LexA regulon in *C. acetobutylicum* contains at least the following genes/operons (Figure 5): *lexA* (CAC1832), *recA* (CAC1815), *uvrB-uvrA*-CAC0504-CAC0505-CAC0506-CAC0507*-uvrC*-CAC0509-*murB* (CAC0502-CAC0510), CAC3343-CAC3344, and *sbcD-sbcC* (CAC2737-CAC2736).

#### Rex: the redox sensor

**Figure 6:**
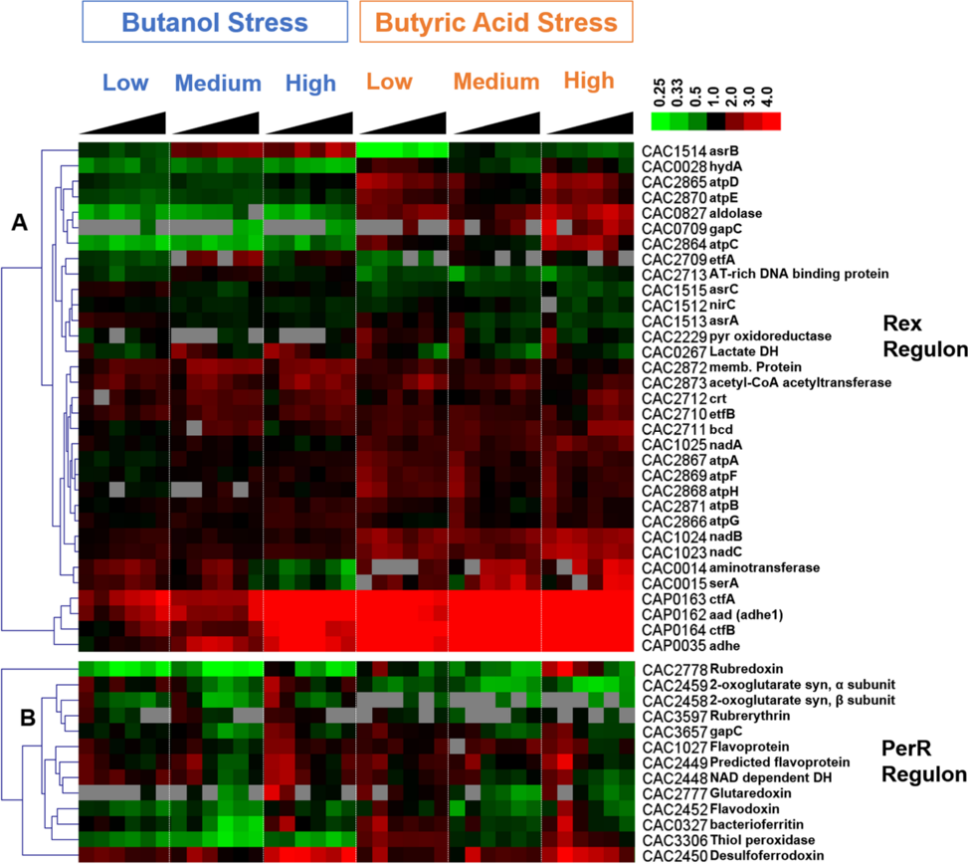
Expression patterns of the predicted Rex (A) and PerR (B) regulons.

Rex (encoded by CAC2713) is a member for the Rex family, which includes regulators that modulate transcription in response to changes in cellular NADH/NAD + levels and more generally the redox state [62]. It was previously demonstrated that Rex is a redox-sensing transcriptional repressor that regulates solventogenesis in *C. acetobutylicum*[63]. In our microarray experiments, *rex* was downregulated under butyrate stress. Our footprinting analysis identified 6 operons with potential Rex binding sites in their promoter regions: *hydA (*CAC0028*), nirC-asrA-asrB-asrC* (CAC1512-CAC1515), *crt-bcd-etfB-etfA* (CAC2712-CAC2709), CAC2713, CAC2873, and *adhE2 (*CAP0035), the fusion-protein aldehyde/alcohol dehydrogenase, which is highly homologous to *adhE1* (CAP0162). In addition, based on orthology to target genes in Rex regulons from RegPrecise and identification of putative Rex binding sites (Matrix Scan with the Rex-binding DNA motif in Clostridiales from RegPrecise, p-value < 1e-3), the following operons were found to be putative targets for Rex regulation in *C. acetobutylicum* as well: CAC0014*-serA* (CAC0014-CAC0015), *ldh* (CAC0267), *gapC* (CAC0709), CAC0827, *nadA-nadB-nadC* (CAC1025-CAC1023), CAC2229, CAC2872*-atpB*-*atpE-atpF-atpH-atpA-atpG-atpD-atpC* (CAC2872-CAC2864), *adhE1-ctfA-ctfB* (CAP0162-CAP0164). The expression patterns for the genes in these operons are shown in Figure 6A. It is noteworthy that *adh*E2 is highly upregulated, together with *adhE1-ctfa-ctfb*. The latter is the set of genes responsible for butanol production in this organism [5]. As discussed above, these genes are also part of Class IV stress response genes.

#### PerR

PerR (encoded by CAC2634) represses the expression of most of the genes involved in oxidative stress response, such as *rbr3A-rbr3B, dfx, rd, nror, fprA1 and fprA2*, which code for reverse rubrerythrins, desulfoferrodoxin, rubredoxin, NADH-rubredoxin oxidoreductase (NROR), and oxygen-reducing flavoproteins, respectively [64,65]. PerR probably acts as a peroxide sensor [65]. By facilitating reactive oxygen species (ROS) scavenging, PerR plays an important role in the oxidative stress defense system in *C. acetobutylicum*, which is an obligate anaerobe. Cells lacking this gene exhibit enhanced aerotolerance and increased H_2_O_2_ resistance. Deletion of *perR* does not affect the intracellular level of iron but increases two-fold that of zinc [64]. Several direct targets of PerR were proposed previously by Hillmann *et al.*, through performing a genome-wide search for potential PerR binding sites using Virtual Footprint software in combination with global transcription analysis of the *perR* deletion mutant against the wild type [65]. Our microarray data for these genes are shown clustered in Figure 6B. Apparently, the Alsaker *et al.* (2010) study showed strong upregulation in these genes for the butyrate stress, but this was not the case in our new study. This may suggest that our new fermentative and culture conditions (with pH control) lead to less oxidative stress and therefore these PerR-regulated genes remained largely repressed or only mildly derepressed.

### Amino- and nucleic-acid transcriptional regulators engaged in the metabolite stress responses

#### ArgR: the arginine repressor

The arginine repressor (ArgR) is known as a master regulator of arginine biosynthesis and catabolism in bacteria in response to intracellular arginine levels [66]. As several genes related to arginine biosynthetic process show strong bimodal expression under both butanol and butyrate stress (Additional file 2: Figure S6), it is of interest to explore the potential involvement of ArgR. ArgR in *C. acetobutylicum* is encoded by CAC2074, the 5^th^ gene in the CAC2078-CAC2073 operon. The ArgR in *E.coli* is known to autoregulate its own transcription [67]. However, it does not appear to be autoregulated in *C. acetobutylicum*, because the promoter region of its operon does not have an identifiable ArgR binding motif (shown in Figure 4A), based on Matrix scan (p-value < 1e-3). Three operons with genes in arginine biosynthetic activity have conserved *cis*-elements in their promoter regions that align well with the known ArgR motifs from RegPrecise (Figure 4A). These operons are *argG-argH* (CAC0973-CAC0974; *argG*, argininosuccinate synthase; *argH*, argininosuccinate lyase), *argB*-*argD* (CAC2389-CAC2388; *argD*, N-acetylornithine aminotransferase; *argB*, acetylglutamate kinase), and *argC-argJ* (CAC2390-CAC2391; *argC*, N-acetyl-gamma-glutamyl-phosphate reductase; *argJ*, ornithine acetyltransferase). Searching for orthologs of the target genes in ArgR regulogs from RegPrecise (a regulog is defined as a set of coregulated genes for which the regulatory sequence has been conserved across multiple organisms [22]) and scanning with the ArgR DNA-binding motif in Clostridiales, several additional operons were identified as potentially also regulated by ArgR. Among them, *argF/I* (CAC0316), *carA* (CAC2645) and CAC3620-CAC3618 (amino acid ABC transporter components) show a more similar expression pattern to those of *argGH, argBD and argCJ* (Figure 4A) than the other genes. Interestingly, this finding suggests that CAC3620-CAC3618 encode a putative arginine transport system, potentially controlling arginine uptake from the environment, as proposed previously for an arginine-responsive gene regulation [68]. Altogether, the ArgR regulon in *C. acetobutylicum* likely includes at least 6 operons/genes (Figure 4A). Furthermore, our *de novo* motif prediction with MOTIFATOR [69] suggests an ArgR binding site upstream of CAC0380. CAC0380 was previously annotated as a periplasmic amino acid-binding protein, but now with our prediction of ArgR binding site for this gene, we suspect it is an arginine-binding protein with a possible role in AR3 mechanism.

#### HisR: histidine biosynthesis regulator

As discussed, histidine biosynthesis genes are dynamically upregulated under both butanol and butyrate stress (Additional file 2: Figure S6). RSAT phylogenetic footprinting [20] analysis shows a well conserved motif upstream of the *hisZ* genes in organisms of the Clostridia class. The *C. acetobutylicum hisZ* gene is in an operon of 10 genes (CAC0935-CAC0944), including *hisZ*, *hisG*, *hisD*, *hisB*, *hisH*, *hisA*, *hisF*, *hisI*, *hisE* and *tkt*. Nine of them show a dynamic upregulation under both butanol and butyrate stress (Figure 4B). The *cis*-elements in the upstream region of *hisZ* genes in Clostridia class (identified by phylogenetic footprinting) bear high similarity to the DNA motif recognized by HisR of *Staphylococcaceae*[22] (Additional file 2: Figure S9). According to OMA [26,70], CAC2675 is an ortholog of *hisR,* which is predicted to be present in several other Gram^+^ organisms in RegPrecise. Based on its orthology to the corresponding target genes assigned in HisR regulogs in RegPrecise and identification of potential HisR-binding site in its promoter region, *hisC* (CAC3031) is also a putative target gene for HisR. To sum, the HisR regulon in *C. acetobutylicum* is proposed to include the *hisZ-tkt* operon and *hisC (*CAC3031*)* (Figure 4B).

#### CymR: cysteine metabolism repressor

In *B. subtilis*, CymR is a master repressor of cysteine metabolism. It controls the expression of genes involved either in cysteine synthesis from sulfide (*cysK*), sulfonates (*ssu*), or methionine (*mccAB*) or in cysteine uptake (*tcyP*) [71]. The activity of CymR is positively regulated by CysK in response to cysteine availability [72]. When cysteine is present, the pool of O-acetylserine (OAS) is low, which leads to the formation of a CymR-CysK complex and transcriptional repression of the CymR regulon occurs. In the absence of cysteine, the OAS pool is high and the CymR-CysK complex is mostly dissociated, leading to a faster dissociation of CymR from its DNA targets and the lifting of CymR-dependent repression [71,72]. Proteins CAC2236 and CAC1675 are the best BLAST hits for the *B. subtilis* CymR [71] in *C. acetobutylicum*. Both proteins belong to the Rrf2 family, with winged helix-turn-helix transcription repressor DNA-binding domain. RegPrecise database assigns CAC1675 as iron-sulfur cluster assembly transcription factor IscR. Indeed, CAC2236 shows slightly higher sequence similarity to CymR than CAC1675 (64.63% vs. 60.69%), whereas CAC1675 has better sequence similarity to IscR in *E. coli* than CAC2236 does (53.09% vs. 49.39%). Therefore it is possible that CAC2236 functions as CymR in *C. acetobutylicum,* and CAC1675 as IscR. In *C. acetobutylicum*, CAC2235, immediately downstream of CAC2236, codes for a cysteine synthase (CysK). In addition, the STRING network for CAC2236 further supports its involvement in regulating cysteine metabolism [73]. On the other hand, RSAT footprinting analysis indicates that CAC1675 has a promoter element conserved in Clostridia class that partially aligns well with the CymR motif from *Staphylococcaceae* (Figure 4C). In fact, the IscR- and CymR-binding DNA motifs share quite significant sequence similarities (Figure 4C) [22]. Furthermore, both CAC2236 and CAC1675 have identifiable CymR-binding sites when searched with the PWM built from TFBSs in *Staphylococcaceae*[22].

Seven operons with potential CymR-binding sites in their promoter regions were identified based on orthology to the CymR target genes in *Bacillales* and *Staphylococcaceae* in RegPrecise, and with pattern matched in promoter regions using Matrix Scan (p-value < 1e-3). They include operons CAC0102-CAC0110 (including genes such as *cysC* (CAC0103), *cysD* (CAC0109), *cysN* (CAC0110); protein CysC and CysN are involved in hydrogen sulfide biosynthetic process) and CAC0878-0880 (amino acid ABC transporter system) (Figure 4C). Making use of the distinct expression pattern of CAC0102-0110 and CAC0878-0880, the other cysteine-metabolism related genes/operons with similar expression pattern, i.e., *cysK* (CAC2235), operons of CAC0929-0931 and CAC3325-3327, were further analyzed for CymR-binding sites. Note that *cysK* and CAC0931 are putatively involved in cysteine biosynthesis from serine and in cysteine synthase activity respectively, whereas CAC3325-CAC3327 encodes protein orthologous to L-cystine-binding protein TcyABC transporter in *Staphylococcus carnosus*. Indeed, *cysK* (CAC2235), CAC0929 and CAC3325 have good match to the CymR-binding motif in their promoter regions (for the latter two, the predicted CymR-binding site is located more than 300 nts upstream their translation start codons). We conclude that the CymR regulon in *C. acetobutylicum* likely includes at least the genes shown in Figure 4C.

#### PurR: repressor of purine metabolism

PurR (encoded by CAC3224) is a member of PurR family, which serves as repressor of purine metabolism [7]. CAC3224 is upregulated under butanol stress, but not so under butyrate stress, which may explain the upregulation of several purine metabolism related genes when under butyrate stress. Interestingly, the majority of *C. acetobutylicum* genes orthologous to the PurR target genes in RegPrecise seem to contain only half of the PurR-binding DNA motif in Bacillales, which is a palindromic sequence comprised of two inverted repeats.

### Complexity of the metabolite stress response as captured by the STRING-based stress response network (SRN)

The high-confidence functional interactions predicted by STRING (Confidence Score ≥ 0.700) were used as edges to construct the butanol/butyrate stress response network (SRN), which is shown in Additional file 2: Figure S8. Cross-interactions are prevalent between most of the key regulons discussed above. Since the majority of the transcription factors and their target genes are highly conserved in a variety of organisms of the *Clostridium* genus (Additional file 4: Table S4), this SRN is likely largely applicable to the other *Clostridium* organisms, In addition, the complexity observed in these two core metabolite stresses may require additional layers of regulation, such as differential mRNA degradation and the regulation at transcriptional and post-transcriptional levels by non-coding small RNAs (sRNAs). The role of sRNA under metabolite stress has been investigated for butanol and butyrate stress based on RNAseq data, and quite a few stress-responsive sRNAs have been identified (Venkataramanan *et al.* 2013, BMC Genomics) [74]. Several sRNAs were particularly up-regulated under both the metabolite stresses. They include 6S RNA (regulating availability of specific sigma factor and hence the expression of genes under their regulation), tmRNA (transfer-messenger RNA, which recycles ribosomes and ensures their availability during a change in the transcriptional and translational machinery of the cells along with labeling aberrant protein for degradation), SRP (signal recognition particle RNA, which regulates the trans-translation of the membrane bound proteins), *solB* (the repressor of the *sol* operon) and SAM riboswitch (regulating the expression of sulfur amino acid metabolism). Identifiable or likely targets of these sRNAs would suggest that these and other sRNAs are likely involved in regulating at least some of the genes in the core regulons discussed in this study.

## Conclusions

Gene regulatory networks play essential roles in living organisms to respond to both external environmental and internal metabolism changes. Understanding the connections and the activity levels of regulators is indispensible for the gene regulatory network research. This study produced a large set of high-resolution temporal transcriptional data. Using phylogenetic footprinting analysis and orthology inference, combined with pattern-based TFBS detection, the regulatory interactions proposed from our study provide important connections between transcription factors and their target genes that are important for butanol and butyrate stress response in *C. acetobutylicum*. Utilizing predicted transcription factor activities in combination with transcriptome data [75] can be a future direction for reconstructing gene regulatory network of *C. acetobutylicum*. It is known that the analysis of transcriptome data does not always permit identifying the primary cause of a phenomenon observed. There are various levels of regulations apart from transcriptional ones, e.g., epigenetic regulation, translational regulations, mRNA stabilities, post-translational modifications. It remains a grand challenge to integrate all these regulation layers together to reconstruct a holistic stress response network. But promising systems biology methods are in development to provide solutions to the problem.

## Methods

### Microarray experiments and data analyses

#### Strain and growth conditions

*C. acetobutylicum* ATCC 824 was grown in a defined clostridial growth medium (40 g/L glucose) in BioFlo®310 Fermentors (New Brunswick Scientific, Edison, NJ) equipped with controllers for pH, temperature, and agitation [12] to an OD_600_ of 1.0 and stressed with appropriate concentrations of butanol (low - 30 mM, med - 60 mM, high - 90 mM), or butyrate (low - 30 mM, med - 40 mM, high - 48 mM). Samples were taken at 6 time points post-stress (0 min, 15 min, 30 min, 45 min, 60 min and 75 min) for RNA isolation. Samples from non-stressed cultures (i.e., 0 mM butanol) were also collected at the same 6 time points, for control. Experiments were carried out in triplicates (2 replicates for preparing labeled-cDNA for microarray hybridization and the third replicate for q-RT-PCR using unlabeled cDNA, for validating the microarray results). All experiments were run in replicates of 4 and the most similar 3 were selected for RNA isolation. The growth curve and product formation patterns are summarized in Additional file 2: Figure S9.

### RNA isolation and labeled cDNA generation

Samples for RNA isolation were collected by centrifuging 15 mL of cultures at 5,000 rpm for 10 min and the cell pellets were frozen at -80°C. Before extracting RNA, the pellets were thawn and RNA was extracted using Qiagen’s RNeasy Mini Kit as described earlier [5]. cDNA generation and its subsequent amino allyl labeling were performed as described [47].

### Microarray analysis

Microarray analysis was performed using Agilent custom arrays (4x44K arrays, GEO accession number GPL10908) by hybridizing 250 ng of Cy3/Cy5 labeled cDNA hybridized against 250 ng oppositely labeled (Cy5/Cy3) common reference pool (containing equal amount of 2.5 μg labeled cDNA from each of the 48 samples) at 65°C for 16–18 hours. Separate reference pools were created for butanol and butyrate stress, respectively. Following hybridization, the slides were washed and scanned in an Agilent scanner; image analysis was carried out using Agilent’s Feature Extraction Software (v9.5.1). Normalization was carried out using the LOESS method in R (Limma package from Bioconductor [76]) using a custom script/algorithm developed in Papoutsakis lab based on SNN-LERN (Segmental Nearest Neighbor normalization method) [77]. Normalized outputs contained averaged, normalized values of replicates and dye-swaps with respect to the common reference pool. The microarray data can be accessed at GEO through the access numbers GSE48031 and GSE48039, respectively.

The data were analyzed by pairwise and point-by-point comparison to the non-stress control using significant analysis of microarrays (SAM) [27] in TIGR MeV suite 4.8.1 [78]. For a gene to be identified as being differentially expressed for a metabolite stress, it had to be significant (5% FDR for SAM analysis) and have at least five time points out of the 18 with expression changes (6 time points for each stress level of Low, Medium and High) over 1.5 fold (up or down) for that metabolite stress. To be considered as bimodally expressed, the gene had to have at least five time points for 1.5 fold up and five time points for 1.5 fold down individually for that metabolite stress. Normalized ratios were grouped by K-means clustering (TIGR MeV, version 4.8.1 [78]) and visualized with heat (or Eisen) plots [79] using TIGR MeV [78]. The K-means clustering was carried out with Euclidean distance metric. We chose K-Means clustering because the number of clusters produced can be directly controlled by the user-defined parameter K without having to somewhat arbitrarily cut a clustering tree like for the case of hierarchical clustering. The microarray data was validated using q-RT-PCR for 6 genes, which were validated for upregulation (CAP0102, CAC1391, CAC1405 & CAC3190), downregulation (CAP0102, CAC0766 & CAC1806), butanol stress (CAP0102, CAC1405, CAC1806 & CAC3190) and butyrate stress (CAP0102, CAC1391 & CAC0766). CAC3571 was used as the house keeping gene. The comparison between Q-RT-PCR and microarrays are summarized in Additional file 2: Figure S10.

#### Transcription factor binding site analysis

DNA sequences up to 300 nt upstream from the start codon of genes in *C. acetobutylicum* were obtained using RSAT Sequence tools. Given a user-provided query gene, RSAT footprint-discovery analysis provides PWMs for over-represented oligonucleotides (words) or spaced pairs thereof (dyads) for the query gene’s orthologs in a user-defined taxon [20]. The obtained PWMs were compared against the PWMs of known TF binding DNA motifs from three resources: RegPrecise [22], RegTransbase [23] and PRODORIC databases [24], with the tool Tomtom in suite MEME [25]. The results, together with OMA ortholog search in *C. acetobutylicum* genome for the predicted TFs and target genes (TGs) defined in RegPrecise [22,26], allowed the inference of TFs and their target genes in *C. acetobutylicum*. RSAT Matrix Scan is used to scan the *C. acetobutylicum* upstream sequences with PWMs to identify instances of the corresponding motifs (putative TFBSs).

### Identification of genes associated with stress responses from the literature and databases

We collected 318 *B. subtilis* stress related proteins through data mining and text mining. With BLAST search between *B. subtilis* and *C. acetobutylicum*, 515 *C. acetobutylicum* proteins matching to 209 out of those 318 *B. subtilis* stress proteins are obtained. We also obtained PPIs from STRING database, a pre-computed database for the exploration of protein-protein interactions (PPIs). The 9.05 version of STRING, which was the newest version at the time of the study used here, covers approximately 5 million proteins from 1133 different organisms [73]. We integrated “Actions” data [which include actions such as inhibition, activation, reaction, catalysis, post-translational modification, binding] with the “evidence” data [which include scores for neighborhood, gene fusion, co-occurrence, co-expression, experiments, databases and text mining respectively, and a combined score] of all *C. acetobutylicum* genes for cytoscape analysis [80].

## Abbreviations

TRNs: Transcriptional regulatory networks; TF: Transcription factor; TFBSs: TF binding sites; TR: Transcriptional regulator; DEGs: Differentially expressed genes; RSAT: Regulatory sequence analysis tools; OMA: Orthology MAtrix; TGs: Target genes; PPI: Protein-protein interaction; qRT-PCR: quantitative real time polymerase chain reaction; SAM: Significance analysis of microarrays; MeV: Multiple experiment viewer.

## Competing interests

The authors declare that they have no competing interests.

## Authors’ contributions

KPV carried out the fermentations, RNA isolations, microarray hybridization, image processing and data normalization. KPV and QW analyzed the data. QW carried out the regulon analyses and other computational analyses. HH, CHW and ETP helped with data analysis and interpretation, and participated in the manuscript preparation. ETP and CHW conceived the study, participated in its design and coordination. All authors read and approved the final manuscript.

## Authors’ information

Qinghua Wang and Keerthi Prasad Venkataramanan: co-first author.

## Supplementary Material

Additional file 1 **Detailed information for the differentially expressed genes.** Click here for file

Additional file 2 Supplemental text and figures. Click here for file

Additional file 3 Detailed information for the 164 significantly differentially expressed transcriptional regulators. Click here for file

Additional file 4 **Conservation of SRN (transcription factors and their target genes) in 5 *Clostridium *** **species.** Click here for file
