# Supplementary material for: Transcription factors and genetic circuits orchestrating the complex, multilayered response of Clostridium acetobutylicum to butanol and butyrate stress
Source: BMC Syst Biol. 2013 Nov 6;7:120. doi: 10.1186/1752-0509-7-120 (PMC3828012; doi:10.1186/1752-0509-7-120)
Supplement: Additional file 2 — Supplemental text and figures. [file 1752-0509-7-120-S2.pdf]

## Supplemental materials

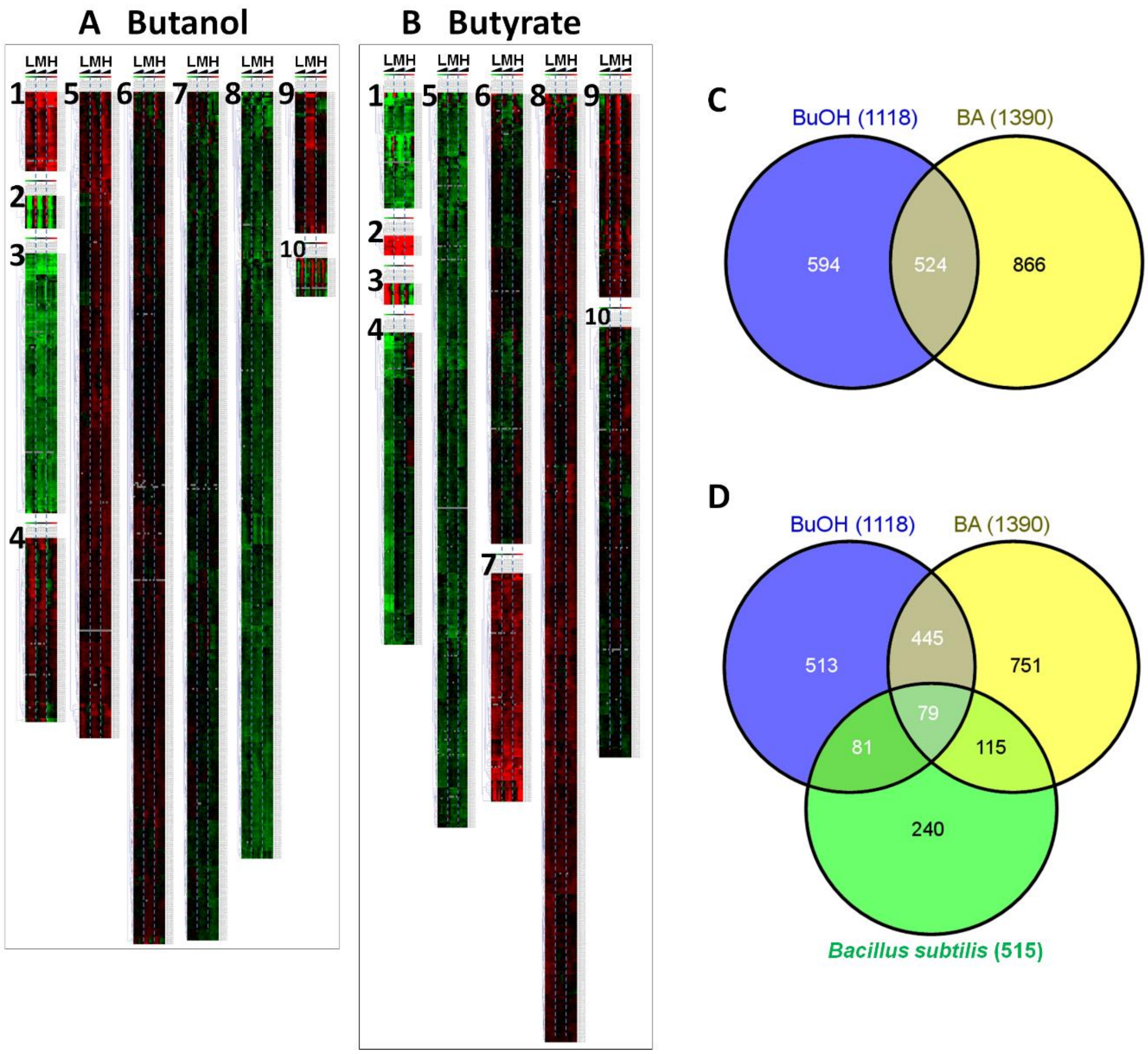


**Fig. S1. Analyses of the microarray expression data**. K-means clustering of 1984 differentially expressed genes under (A) butanol stress and (B) butyrate stress.. For the butanol stress data in (A), cluster 1 is enriched with genes relevant to protein folding and histidine biosynthesis; cluster 2 is enriched with arginine biosynthesis related genes; cluster 5 includes 7 genes involved in aromatic amino acid family biosynthesis, and 6 genes in branched-chain amino acid biosynthesis.  For the butyrate stress (B), cluster 1 is enriched with genes related to sulfur

compound metabolic processes, and inorganic ion transport and metabolism; cluster 2 includes many Class III and IV HSPs; cluster 3 has genes involved in arginine biosynthesis; cluster 5 is enriched with genes for structural constituents of ribosomes, and cobalamin biosynthesis; cluster 7 has 8 genes involved in purine ribonucleoside monophosphate biosynthesis, and several Class I HSPs; cluster 9 is enriched with genes involved in histidine biosynthesis, and carbohydrate transport and metabolism.  (C) Venn diagram of the distribution of the differentially expressed genes between the two conditions (BuOH – Butanol and BA- Butyrate). (D) Comparison of the differentially expressed genes with the 515 genes with certain homology to *B. subtilis* stress-related proteins (see manuscript Methods section for details).

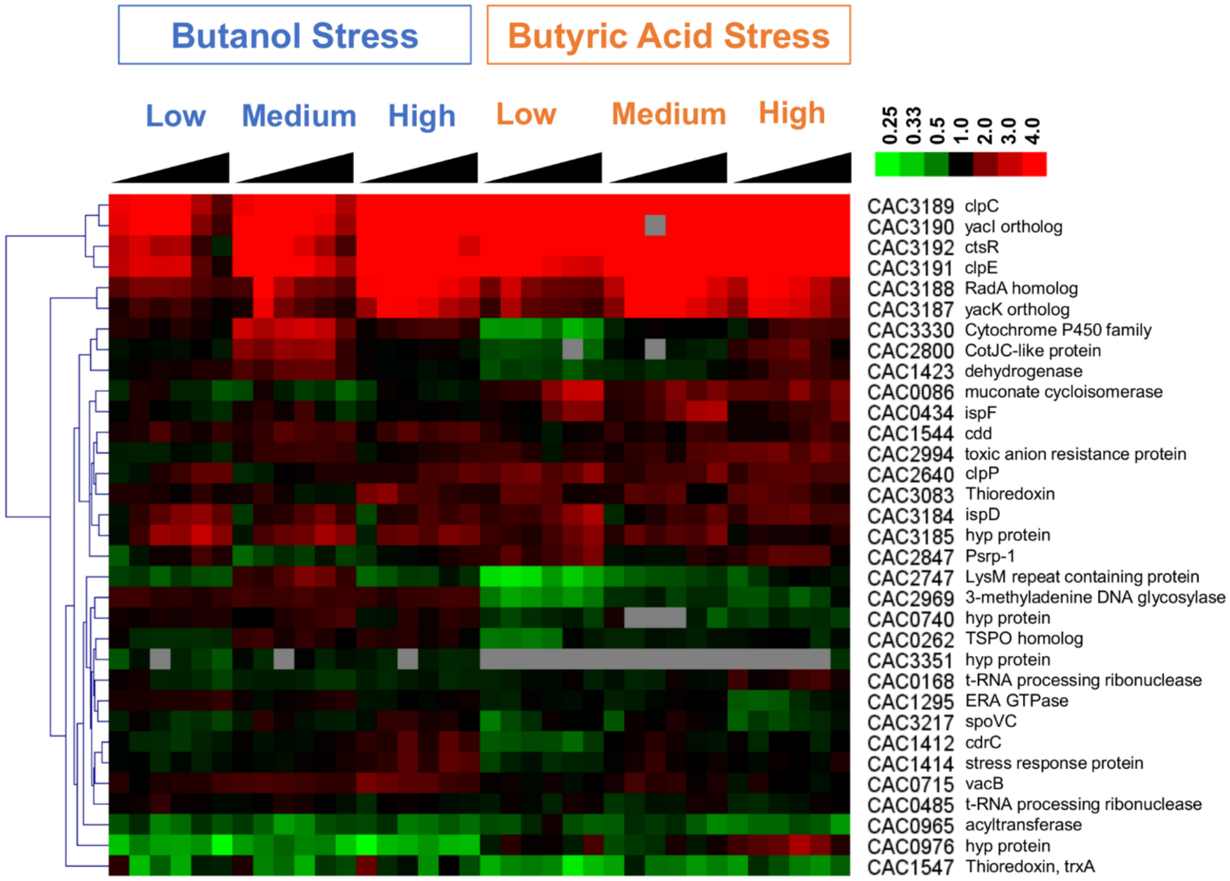


**Fig. S2**. Orthologs of *B. subtilis* Class II HSPs in *C. acetobutylicum* based on orthology analysis (OMA [1, 2]). Genes CAC3187-CAC3192 belong to Class III HSPs regulated by CtsR and the other genes show no significant differential expression under stress, which confirms the absence of a functional $\sigma^B$ in *C. acetobutylicum*.

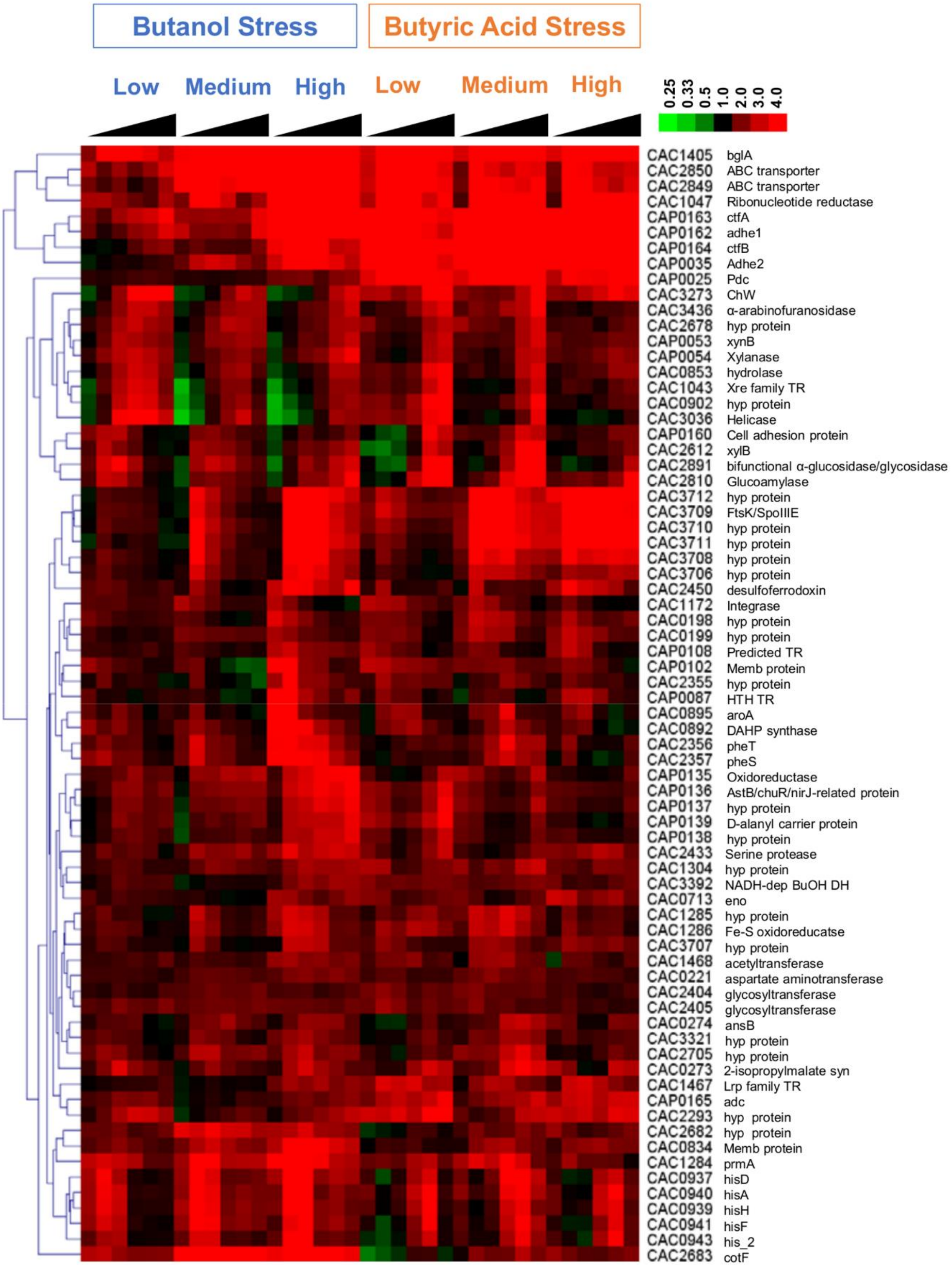


**Fig. S3**. Class IV stress genes in *C. acetobutylicum* based on their upregulation under both butanol and butyrate stress, and by excluding genes regulated by either HrcA or CtsR.

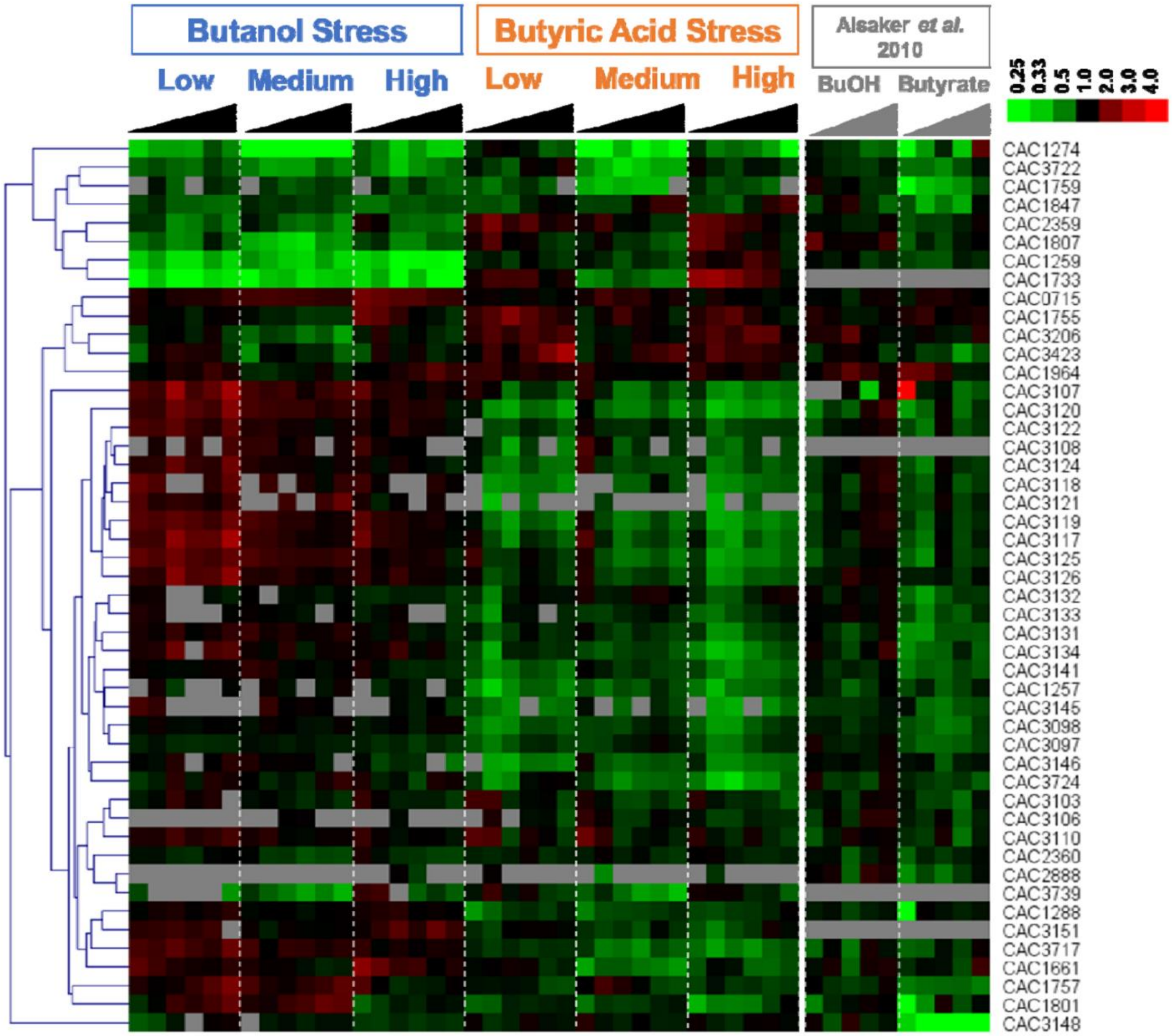


**Fig. S4**. Hierarchical clustering of 48 ribosomal proteins in *C. acetobutylicum* under butanol and butyrate stress and comparison with the data from Alsaker *et al.* 2010 study [3].

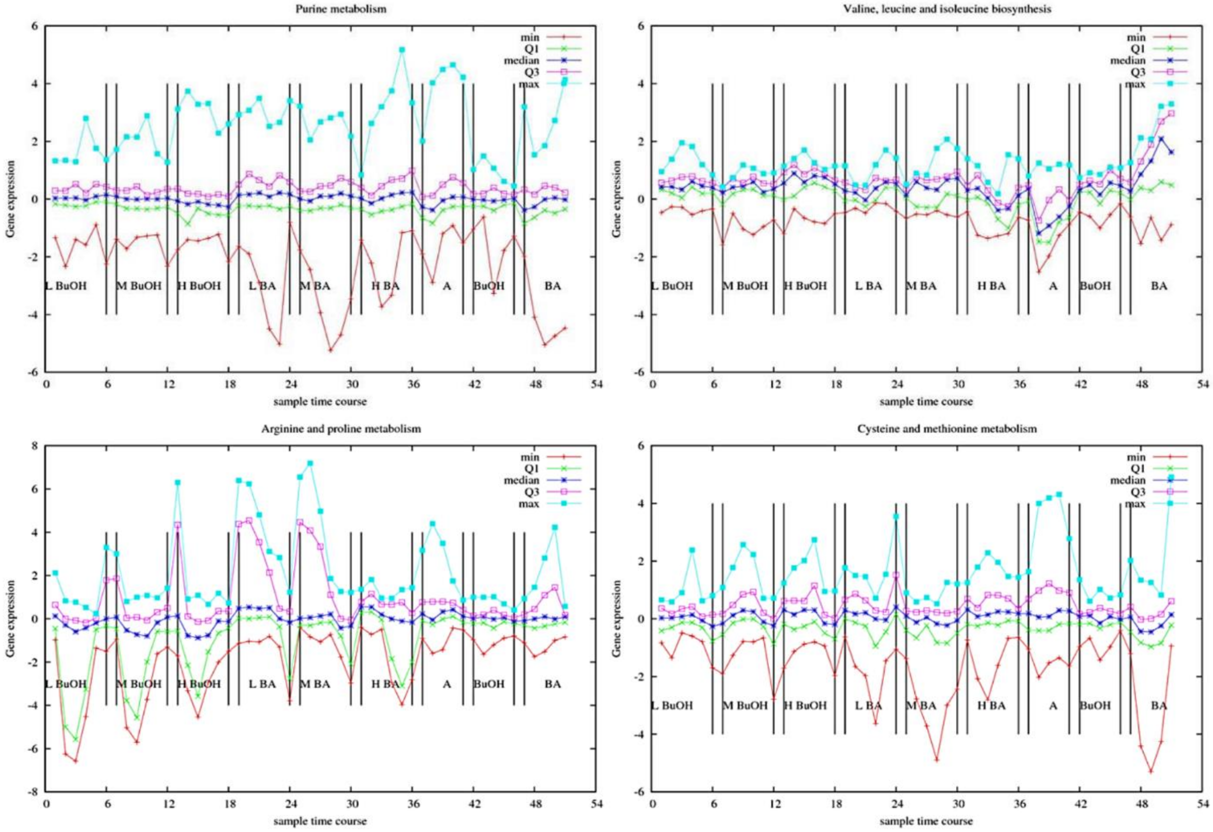


**Fig. S5.** The so-called 5-number summary (i.e., the median, the upper and lower quartiles, and the minimum and maximum) of a dataset for the logarithmic (to the basis of 2) expression ratios under this study (i.e., BuOH L(ow) M(edium) H(igh), BA L M H, at the tested time points) and in the 2010 Alsaker study (i.e. “A”, “BuOH”, “BA”) for the genes of four KEGG pathways in *Clostridium acetobutylicum*. The x-axis is indexing a sample (e.g., the sample L BuOH at time 0 min is shown at 1 for x-axis, whereas L BA at time 0 min is shown at 19). The y-axis is the logarithm of gene expression ratios. The four panels include purine metabolism (top left, 58 genes), branched chain amino acid (ILV) biosynthesis (Top right, 13 genes); arginine and proline metabolism (bottom left, 23 genes); and cysteine and methionine metabolism (bottom right, 32 genes). An interesting finding is that, frequently, not all genes in a given pathway show consistent expression patterns. For example, it is not uncommon that only few genes in a

pathway are strongly differentially expressed, whereas the majority is not. For example, among the 58 *C. acetobutylicum* genes involved in purine metabolism (upper left panel), most do not experience a significant change in expression under stress (i.e., their logarithmic expression ratios are close to 0). This holds true also for the valine/leucine/isolucine biosynthesis and cysteine/methionine metabolism pathways as displayed in these graphs. These data suggest that a pathway could be regulated by changing the expression of a subset of its genes, rather than the whole gene set, and this is consistent with the well-established notion of the rate- or process controlling genes, enzymes or reactions. On the other hand, argine/proline metabolism pathway is an example for pathways with a significant subset of genes consistently differentially expressed (lower left panel).

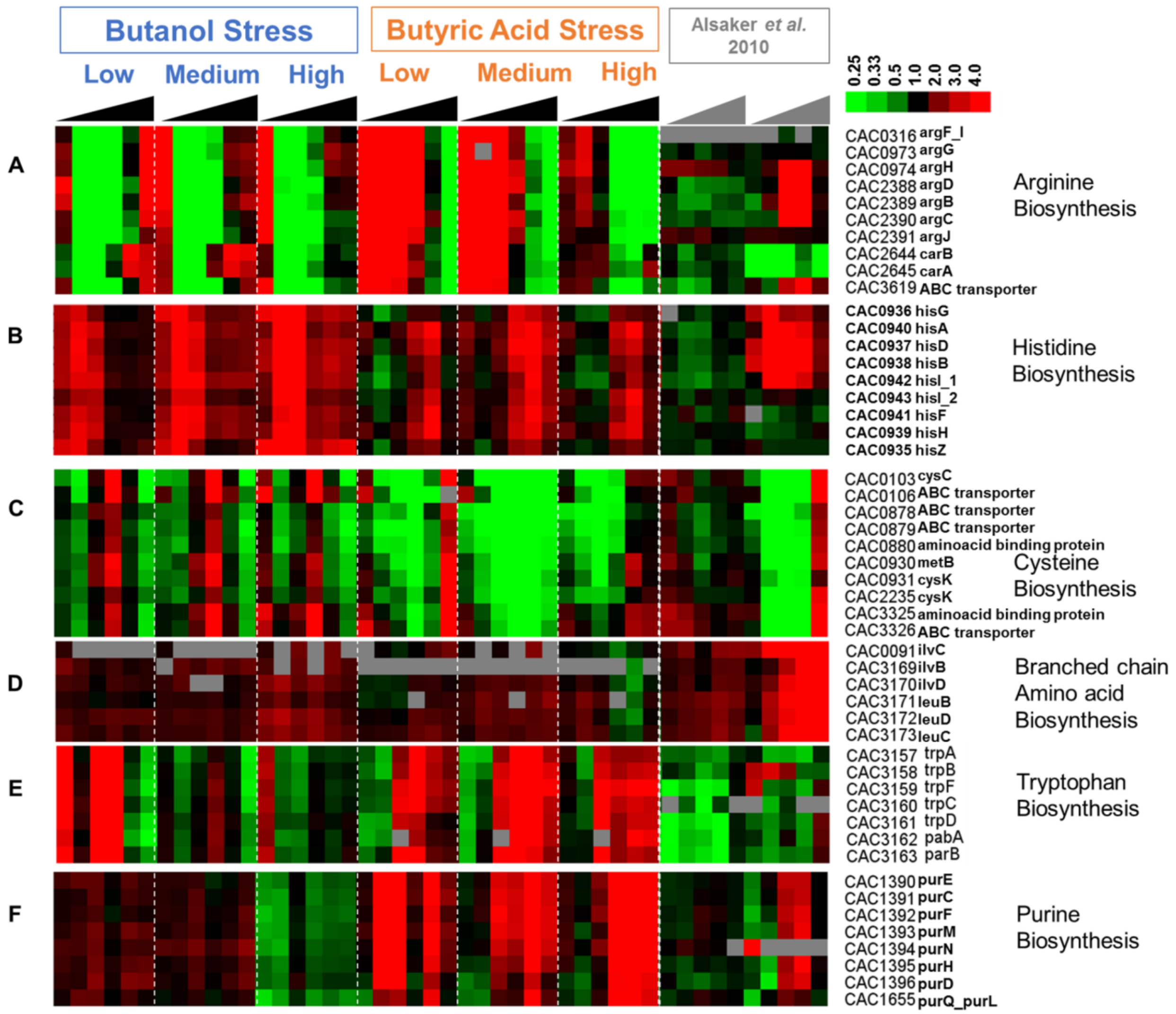
Butanol Stress
Low Medium High
Butyric Acid Stress
Low Medium High
Alsaker et al. 2010
0.25 0.33 0.5 1.0 2.0 3.0 4.0
A
B
C
D
E
F
CAC0316 argF_I
CAC0973 argG
CAC0974 argH
CAC2388 argD
CAC2389 argB
CAC2390 argC
CAC2391 argJ
CAC2644 carB
CAC2645 carA
CAC3619 ABC transporter
Arginine Biosynthesis
CAC0936 hisG
CAC0940 hisA
CAC0937 hisD
CAC0938 hisB
CAC0942 hisI_1
CAC0943 hisI_2
CAC0941 hisF
CAC0939 hisH
CAC0935 hisZ
Histidine Biosynthesis
CAC0103 cysC
CAC0106 ABC transporter
CAC0878 ABC transporter
CAC0879 ABC transporter
CAC0880 aminoacid binding protein
CAC0930 metB
CAC0931 cysK
CAC2235 cysK
CAC3325 aminoacid binding protein
CAC3326 ABC transporter
Cysteine Biosynthesis
CAC0091 ilvC
CAC3169 ilvB
CAC3170 ilvD
CAC3171 leuB
CAC3172 leuD
CAC3173 leuC
Branched chain Amino acid Biosynthesis
CAC3157 trpA
CAC3158 trpB
CAC3159 trpF
CAC3160 trpC
CAC3161 trpD
CAC3162 pabA
CAC3163 parB
Tryptophan Biosynthesis
CAC1390 purE
CAC1391 purC
CAC1392 purF
CAC1393 purM
CAC1394 purN
CAC1395 purH
CAC1396 purD
CAC1655 purQ_purL
Purine Biosynthesis

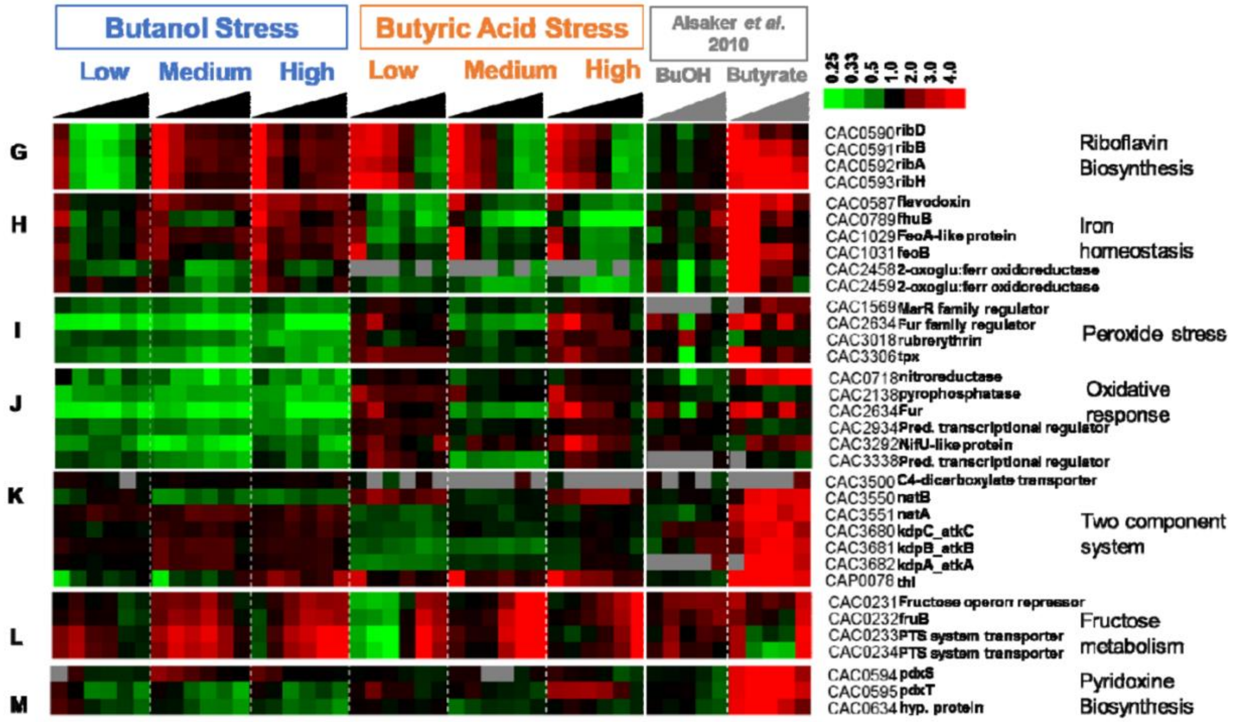


**Fig. S6**. Comparisons on selected genes between this study and the previous study by Alsaker et al. in 2010 [3].

(A) 10 genes in Arg biosynthesis.
(B) 9 genes in His metabolism.
(C) 10 genes in Cys metabolism.
(D) 6 genes in Ile, Leu, Val biosynthesis.
(E) 7 genes in Trp biosynthesis
(F) 8 genes in Purine metabolism.
(G) 4 genes in Riboflavin Biosynthesis
(H) 6 genes involved in iron homeostasis show strong difference in response to 30mM butyrate stress.
(I) 4 genes in Peroxide stress
(J) 6 genes in Oxidative response
(K) 7 genes related to two-component system
(L) 4 genes in Fructose metabolism
(M) 3 genes in Pyridoxine metabolism

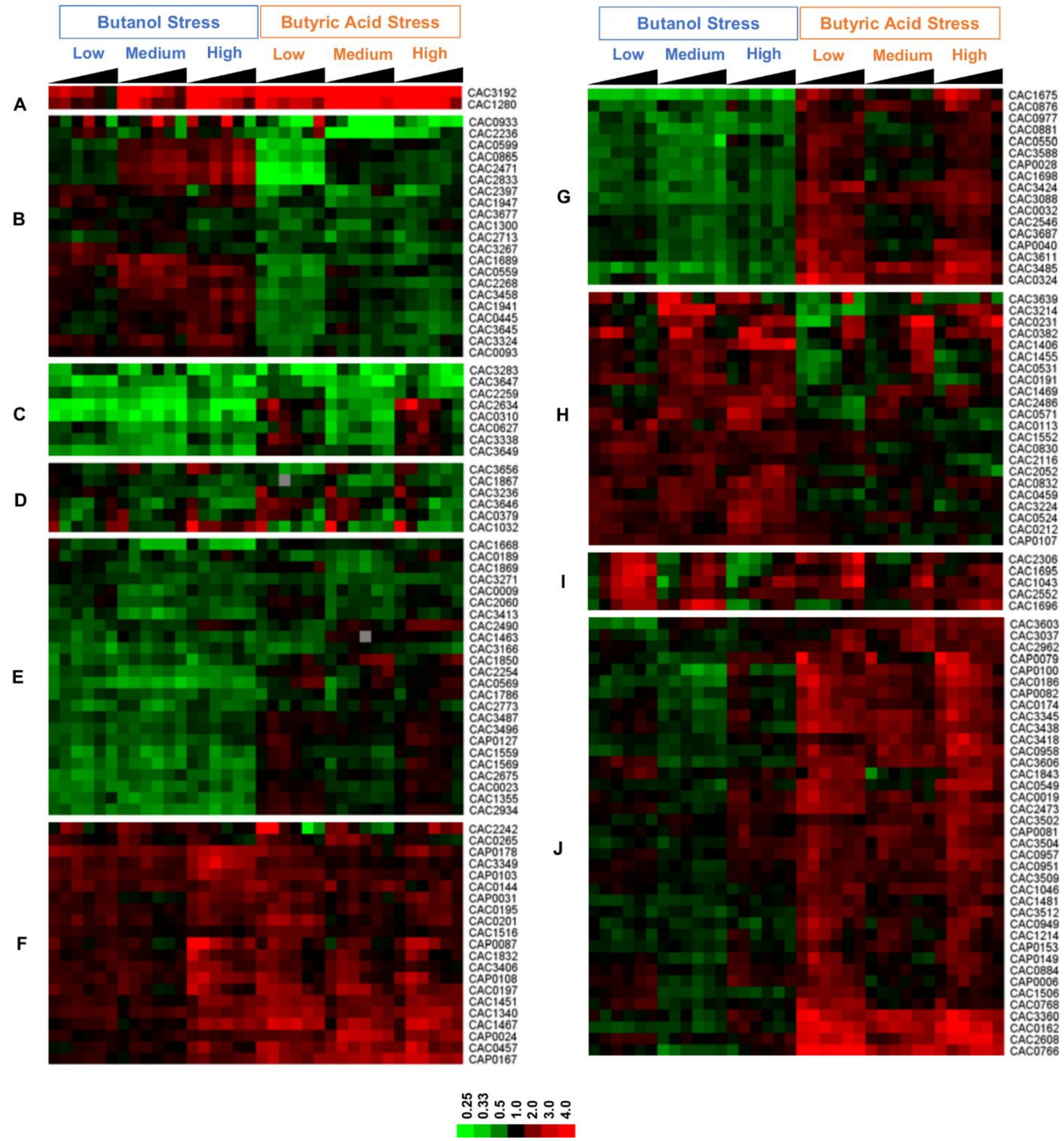


**Fig. S7.** K-means clustering into 10 user-defined clusters of 164 differentially expressed transcriptional regulators in C. acetobutylicum under butanol and butyrate stress. Clusters A, F & I represent upregulated TRs, while C, D & E contain downregulated TRs (general stress response). On the other hand, clusters B, G, H & J consist of TRs with distinct expression patterns for each stress and are involved in orchestrating the stressor-specific response.

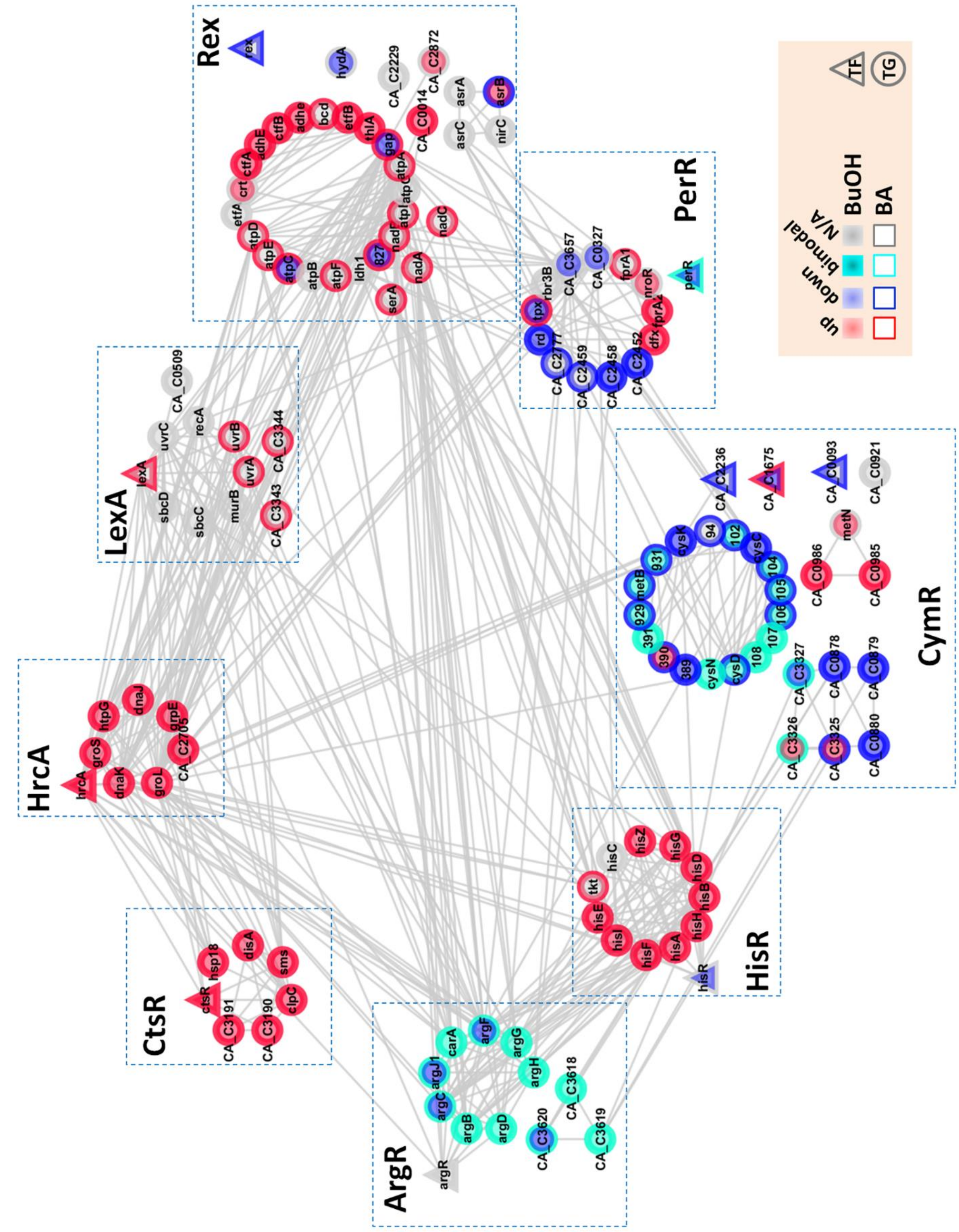


**Fig. S8.** The Stress Response Network (SRN) displaying the inter-relationships among the eight core, stress-related, regulons.

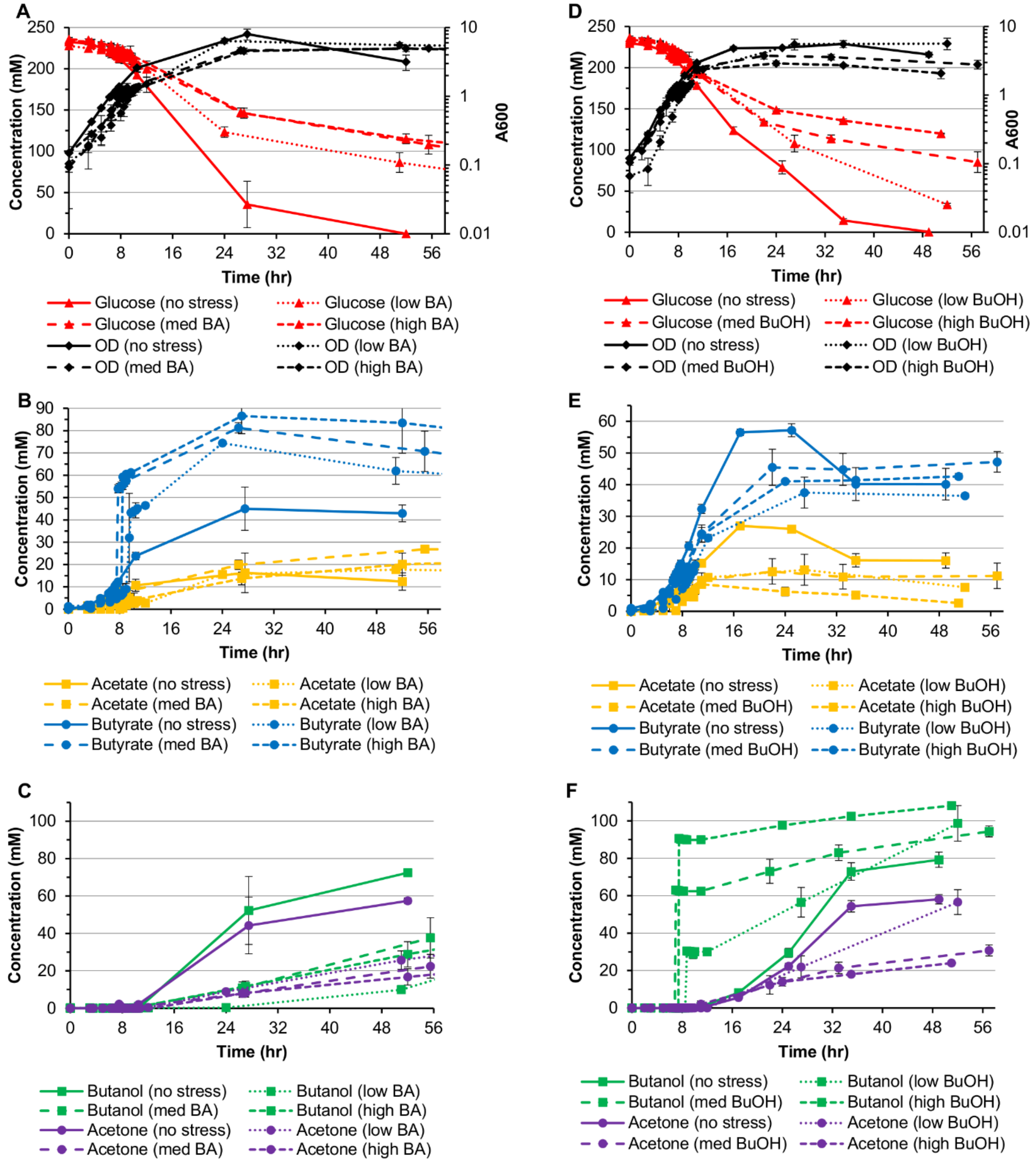


**Fig. S9**. Growth curve for butyrate stress (A) and butanol stress (D). Product formation for butyrate stress (B & C) and butanol stress (E & F).

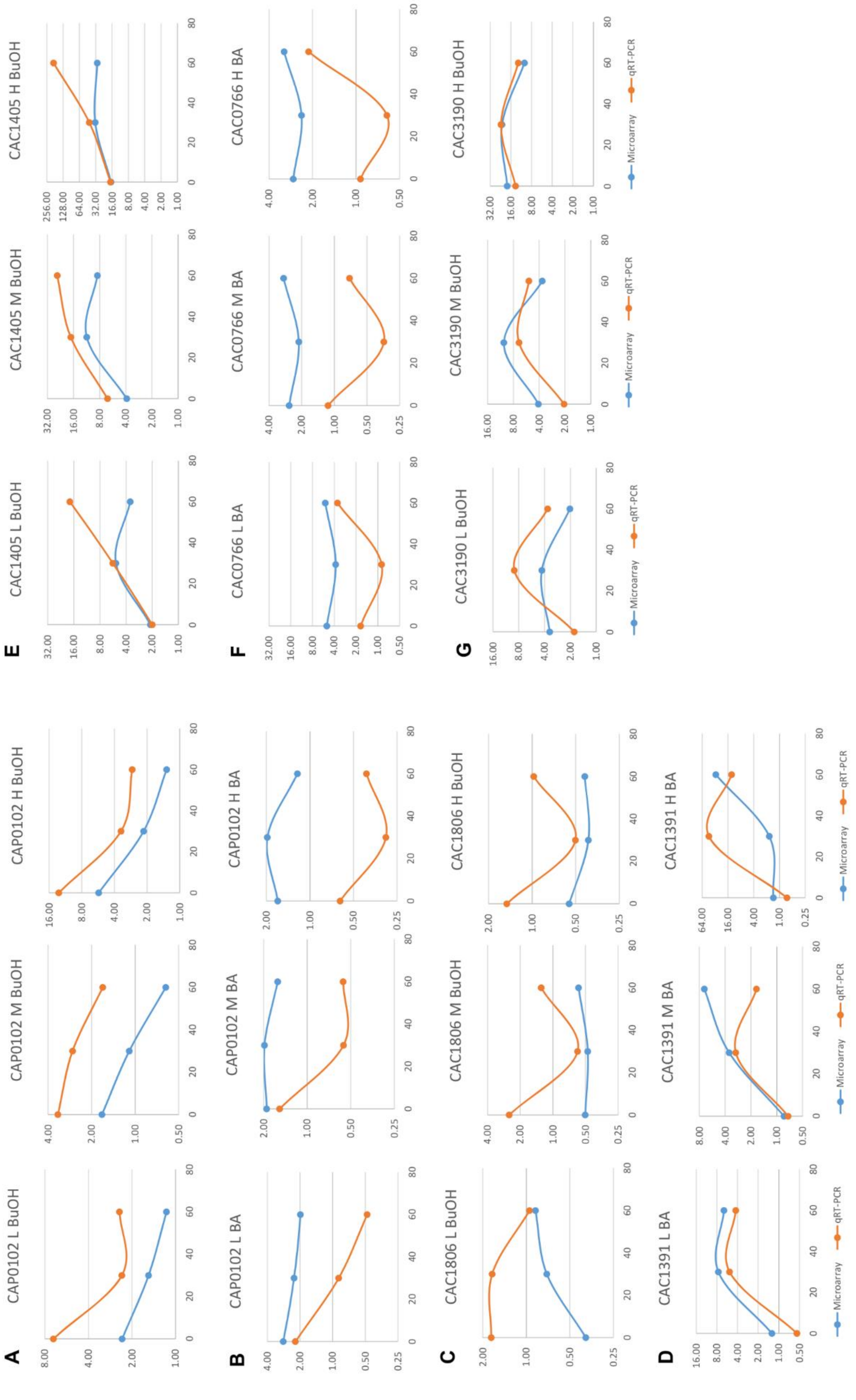
A
CAP0102 L BuOH
CAP0102 M BuOH
CAP0102 H BuOH
B
CAP0102 L BA
CAP0102 M BA
CAP0102 H BA
C
CAC1806 L BuOH
CAC1806 M BuOH
CAC1806 H BuOH
D
CAC1391 L BA
CAC1391 M BA
CAC1391 H BA
E
CAC1405 L BuOH
CAC1405 M BuOH
CAC1405 H BuOH
F
CAC0766 L BA
CAC0766 M BA
CAC0766 H BA
G
CAC3190 L BuOH
CAC3190 M BuOH
CAC3190 H BuOH
Microarray
qRT-PCR

**Fig. S10**. Comparison of microarray (blue points and lines) against Q-RT-PCR (orange points and lines). A. CAP0102 – butanol (BuOH) stress; B. CAP0102 – butyrate (BA) stress; C. CAC1806; D. CAC1391; E. CAC1405; F. CAC0766; G. CAC3190.

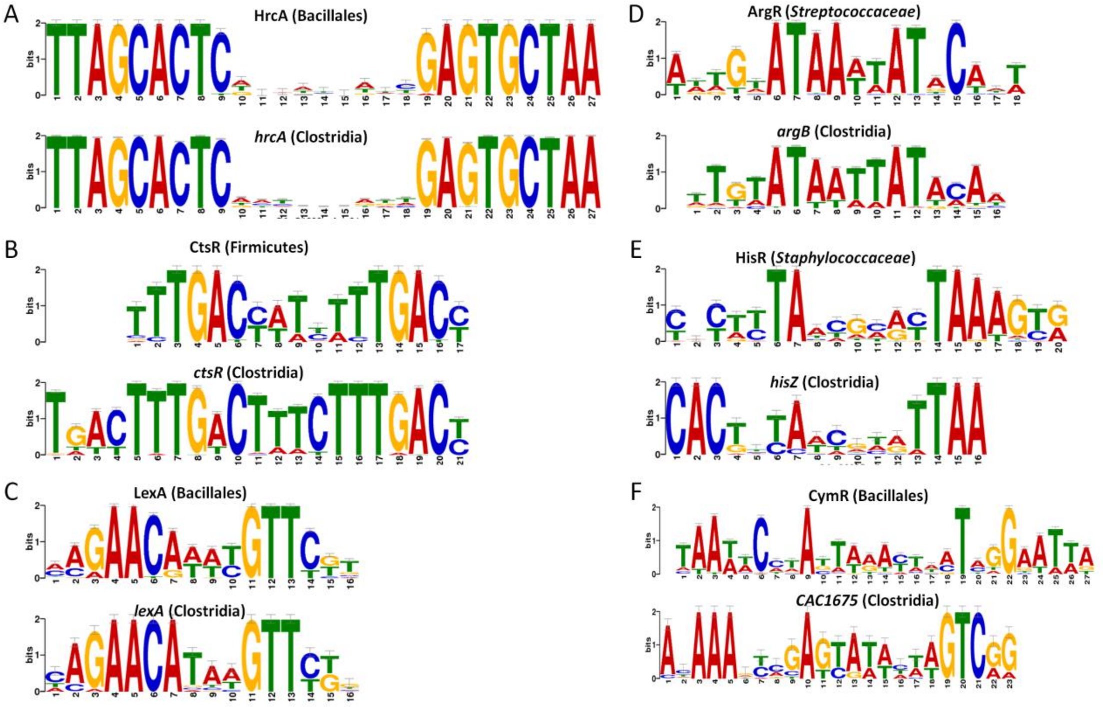


**Fig. S11**. Comparison of the DNA motifs for the binding of (A) HrcA, (B) CtsR, (C) LexA, (D) ArgR, (E) HisR and (F) CymR from RegPrecise [4] (the upper sequence logos in each panel) with those for the identified *cis*-elements in the class of Clostridia for the given genes, i.e., *hrcA*, *ctsR*, *lexA*, *argB*, *hisZ* and CAC1675 (the lower sequence logos in each panel).

## Reference

1. Schneider, A., C. Dessimoz, and G.H. Gonnet, *OMA Browser - Exploring orthologous relations across 352 complete genomes.* Bioinformatics, 2007. **23**(16): p. 2180-2182.
2. Altenhoff, A.M., et al., *OMA 2011: orthology inference among 1000 complete genomes.* Nucleic Acids Research, 2011. **39**: p. D289-D294.
3. Alsaker, K.V., C. Paredes, and E.T. Papoutsakis, *Metabolite stress and tolerance in the production of biofuels and chemicals: gene-expression-based systems analysis of butanol, butyrate, and acetate stresses in the anaerobe Clostridium acetobutylicum.* Biotechnol Bioeng, 2010. **105**(6): p. 1131-47.
4. Novichkov, P.S., et al., *RegPrecise: a database of curated genomic inferences of transcriptional regulatory interactions in prokaryotes.* Nucleic Acids Research, 2009. **38**(Database): p. D111-D118.
